# Recent genetic connectivity and clinal variation in chimpanzees

**DOI:** 10.1038/s42003-021-01806-x

**Published:** 2021-03-05

**Authors:** Jack D. Lester, Linda Vigilant, Paolo Gratton, Maureen S. McCarthy, Christopher D. Barratt, Paula Dieguez, Anthony Agbor, Paula Álvarez-Varona, Samuel Angedakin, Emmanuel Ayuk Ayimisin, Emma Bailey, Mattia Bessone, Gregory Brazzola, Rebecca Chancellor, Heather Cohen, Emmanuel Danquah, Tobias Deschner, Villard Ebot Egbe, Manasseh Eno-Nku, Annemarie Goedmakers, Anne-Céline Granjon, Josephine Head, Daniela Hedwig, R. Adriana Hernandez-Aguilar, Kathryn J. Jeffery, Sorrel Jones, Jessica Junker, Parag Kadam, Michael Kaiser, Ammie K. Kalan, Laura Kehoe, Ivonne Kienast, Kevin E. Langergraber, Juan Lapuente, Anne Laudisoit, Kevin Lee, Sergio Marrocoli, Vianet Mihindou, David Morgan, Geoffrey Muhanguzi, Emily Neil, Sonia Nicholl, Christopher Orbell, Lucy Jayne Ormsby, Liliana Pacheco, Alex Piel, Martha M. Robbins, Aaron Rundus, Crickette Sanz, Lilah Sciaky, Alhaji M. Siaka, Veronika Städele, Fiona Stewart, Nikki Tagg, Els Ton, Joost van Schijndel, Magloire Kambale Vyalengerera, Erin G. Wessling, Jacob Willie, Roman M. Wittig, Yisa Ginath Yuh, Kyle Yurkiw, Klaus Zuberbuehler, Christophe Boesch, Hjalmar S. Kühl, Mimi Arandjelovic

**Affiliations:** 1 Max Planck Institute for Evolutionary Anthropology (MPI EVAN), Leipzig, Germany; 2 German Centre for Integrative Biodiversity Research (iDiv) Halle-Jena-Leipzig, Leipzig, Germany; 3 Jane Goodall Institute Spain and Senegal, Dindefelo Biological Station, Dindefelo, Kedougou Senegal; 4 West Chester University, Depts of Anthropology & Sociology and Psychology, West Chester, PA USA; 5 Department of Wildlife and Range Management, Faculty of Renewable Natural Resources, Kwame Nkrumah University of Science and Technology, Kumasi, Ghana; 6 WWF Cameroon Country Programme Office, Yaoundé, Cameroon; 7 Chimbo Foundation, Amsterdam, Netherlands; 8 Elephant Listening Project, Center for Conservation Bioacoustics, Cornell Lab of Ornithology, Cornell University, Ithaca, NY USA; 9 Department of Social Psychology and Quantitative Psychology, Faculty of Psychology, University of Barcelona, Barcelona, Spain; 10 Biological and Environmental Sciences, Faculty of Natural Sciences, University of Stirling, Stirling, UK; 11 University of Cambridge, Cambridge, UK; 12 Wild Chimpanzee Foundation (WCF), Leipzig, Germany; 13 School of Human Evolution and Social Change, Arizona State University, 900 Cady Mall, Tempe, AZ 85287 Arizona State University, Tempe, AZ USA; 14 Comoé Chimpanzee Conservation Project, Comoé National Park, Kakpin, Côte d’Ivoire; 15 Ecohealth Alliance, New York, NY USA; 16 University of Antwerp, Campus Drie Eiken, lokaal D.133, Universiteitsplein 1 - 2610, Antwerpen, Belgium; 17 Agence National des Parcs Nationaux (ANPN) Batterie 4, Libreville, Gabon; 18 Ministère des Eaux, des Forêts, de la Mer, de l’Environnement, Chargé du Plan Climat, des Objectifs de Développement Durable et du Plan d’Affectation des Terres, Libreville, Gabon; 19 Lester E. Fisher Center for the Study and Conservation of Apes, Lincoln Park Zoo, Chicago, IL USA; 20 Budongo Conservation Field Station, Masindi, Uganda; 21 Panthera, New York, NY USA; 22 Department of Anthropology, University College London, London, UK; 23 West Chester University, Department of Psychology, West Chester, PA USA; 24 Washington University in Saint Louis, Department of Anthropology, One Brookings Drive, St. Louis, MO USA; 25 Wildlife Conservation Society, Congo Program, Brazzaville, Republic of Congo; 26 National Protected Area Authority, Freetown, Sierra Leone; 27 School of Biological & Environmental Sciences, Liverpool John Moores University, Liverpool, UK; 28 KMDA, Centre for Research and Conservation, Royal Zoological Society of Antwerp, Antwerp, Belgium; 29 Department of Human Evolutionary Biology, Harvard University, Cambridge, MA USA; 30 Taï Chimpanzee Project, Centre Suisse de Recherches Scientifiques, Abidjan, Côte d’Ivoire; 31 Pan Verus Project Outamba-Kilimi National Park, Freetown, Sierra Leone; 32 Université de Neuchâtel, Institut de Biologie, Neuchâtel, Switzerland; 33 School of Psychology and Neuroscience, University of St Andrews, St Andrews, UK

**Keywords:** Structural variation, Evolutionary biology, Classification and taxonomy, Evolutionary ecology, Genotyping and haplotyping

## Abstract

Much like humans, chimpanzees occupy diverse habitats and exhibit extensive behavioural variability. However, chimpanzees are recognized as a discontinuous species, with four subspecies separated by historical geographic barriers. Nevertheless, their range-wide degree of genetic connectivity remains poorly resolved, mainly due to sampling limitations. By analyzing a geographically comprehensive sample set amplified at microsatellite markers that inform recent population history, we found that isolation by distance explains most of the range-wide genetic structure of chimpanzees. Furthermore, we did not identify spatial discontinuities corresponding with the recognized subspecies, suggesting that some of the subspecies-delineating geographic barriers were recently permeable to gene flow. Substantial range-wide genetic connectivity is consistent with the hypothesis that behavioural flexibility is a salient driver of chimpanzee responses to changing environmental conditions. Finally, our observation of strong local differentiation associated with recent anthropogenic pressures portends future loss of critical genetic diversity if habitat fragmentation and population isolation continue unabated.

## Abstract (web summary)

Lester and colleagues use faecal samples and genetic analyses to investigate the genetic connectivity across chimpanzees. Their results indicate that the global pattern of genetic diversity in chimpanzees is largely characterized by a pattern of isolation by distance with several isolated populations exhibiting strong local differentiation.

## Introduction

Humans have been characterized as a genetically continuous species^1^, which is expected to hamper local adaptation^2,3^. Our species has largely relied on behavioural flexibility^4^ to become among the most widely distributed species, inhabiting a diverse range of climates and habitats^5^. Among our closest living relatives, the chimpanzee (*Pan troglodytes*)^6^, also exhibits extensive behavioural variation, both at a local and regional scale, and occupies a broad range of habitats and climates, while displaying little associated morphological variation^7,8^. However, to date, their pattern of genetic diversity is equivocal: some studies provide evidence of connectivity among all populations^9,10^, while others have concluded that chimpanzees are taxonomically divided into four geographical subspecies^11–15^. Given the high degree of behavioural variability and flexibility across chimpanzee populations, characterizing range-wide patterns of genetic diversity in chimpanzees is important for understanding how they adapt to changing environmental conditions^16^. In particular, a signal of genetic connectivity across their range would suggest that, like in humans, local trait fixation is relatively slow in chimpanzees, and behavioural flexibility allows them to quickly and dynamically respond to ecological challenges.

**Fig. 1:**
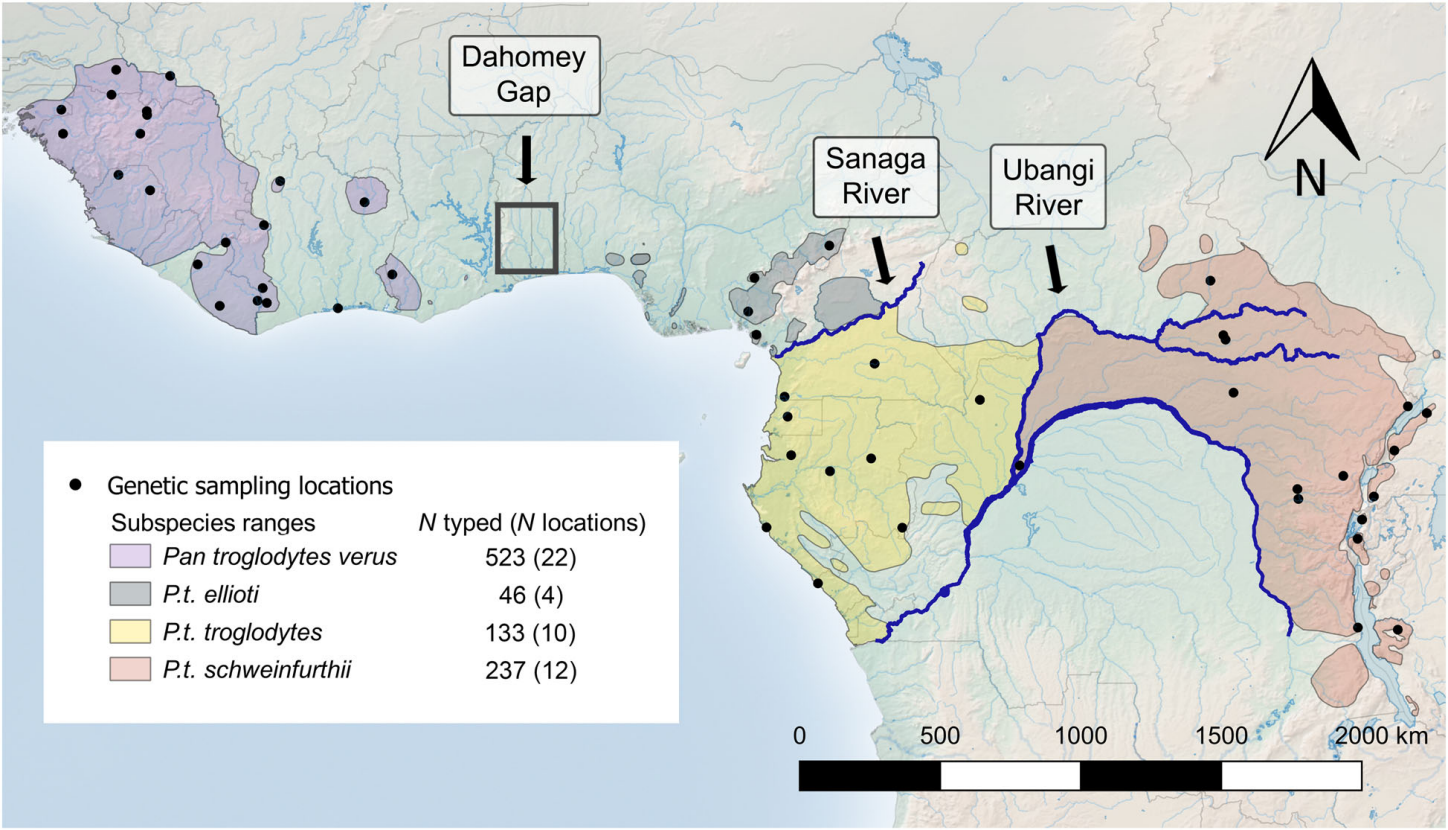
Distribution map of *P. troglodytes* and PanAf sampling. The current approximate chimpanzee subspecies ranges^1^, sample collection locations and proposed subspecies geographic barriers. Total number of genotyped individuals and number of sampling locations are listed for each subspecies population. Much of the historical population located between *P. t. ellioti* and *P. t. verus* populations have been extirpated, creating an extensive sampling gap in the data. Note, samples collected during nationwide studies in Liberia and Equatorial Guinea were included in our spatially explicit analyses and are indicated here as the geographic centre points of the sampling distribution.

Prior to the recent growth and expansion of human agriculturalist settlements across Africa (ca. 5–2 ka BP)^17,18^ chimpanzees were nearly continuously distributed across Equatorial Africa^7^. Rivers appear to be the main barriers to migration^7^ separating chimpanzees and bonobos (*Pan paniscus*), as well as three of the four currently recognized chimpanzee subspecies: Nigeria–Cameroon (*Pan troglodytes ellioti*), central (*Pan troglodytes troglodytes*) and eastern (*Pan troglodytes schweinfurthii*) chimpanzees^19^ (Fig. 1). River systems, however, are dynamic and may become permeable to dispersal during arid periods, or when natural bridges form^20^. Moreover, the presence of this type of barrier does not preclude the possibility of gene flow occurring around it, which has not been tested on the subspecies-delineating barriers in any previous studies of chimpanzees. Similarly, the Dahomey Gap, an arid, 200-km-wide forest-savannah mosaic that separates western chimpanzees (*Pan troglodytes verus*) and *P. t. ellioti*, is also dynamic, as this region hosted rainforest as recently as 4 ka BP (ref. ^21^; ~160 chimpanzee generations^22^). Interestingly, discrete geographic clustering of morphological traits along subspecies lines has not been conclusively shown^23–25^ and, although chimpanzees do show substantial behavioural variation across populations, to date, there are no universal subspecies-specific behaviours, i.e., accumulative stone throwing is unique to *P. t. verus*, but has only been observed at a few study sites^26^.

In a continuously distributed population, limited dispersal drives a predictable pattern of genetic diversity, known as isolation by distance (IBD), which manifests as a continuous gradient (cline) of declining similarity as geographical distance increases^27^. Detectable departures from this pattern are evidence of geographic or behavioural barriers that reduce or impede dispersal, thereby leading to increased geographical rates of genetic differentiation. Although chimpanzees are thought to have had a historically continuous geographic distribution across their range^7^, studies of chimpanzee genetic diversity have tended to show discontinuities consistent with the subspecies classifications^11–13^, or some degree of population structuring^14^. To detect geographic clustering of genetic data, these studies relied on several different ‘spatially agnostic’ approaches not accounting for the spatial distribution of samples. These include Bayesian clustering algorithms (e.g., STRUCTURE^28^), the results of which can be biased when IBD is present in the data^29–31^ and principal component analyses, a method specifically intended to maximize between-group differences^32^. However, to date no studies of species-wide chimpanzee population structure have featured spatially explicit methods, which incorporate the geographic location of the genetic samples into the analysis model and assume IBD in the null model^9,33^. Importantly, when using spatially agnostic methods in the presence of IBD, unbalanced sampling will detect discrete stratification of the data, regardless of the actual pattern^9,29,30,33–36^, and this cannot be compensated for by analyzing more loci (i.e., a large number of SNPs)^37^. Mostly due to the logistical challenge of obtaining non-invasive samples from chimpanzees’ wide range, which largely encompasses politically unstable or remote regions, previous studies of chimpanzee population structure have relied on small and clustered datasets, and often made use of zoo and sanctuary samples of unknown provenance, which introduced further uncertainty^11–15^. Therefore, previous reports of chimpanzee population structure were likely biased by applying spatially agnostic analyses to datasets composed of dispersed and uneven sampling in the presence of IBD^9,33^, and were possibly further confounded by incorrect knowledge about populations of origin in some samples. In fact, even the most comprehensive studies of chimpanzee population history, which utilized genome-wide sequence data^14,15^, were based on a relatively small number of samples from captive individuals, and focused more on fitting models of divergence among subspecies than investigating recent genetic connectivity in space.

In this study, we aimed at assessing recent genetic connectivity across the chimpanzees range by analyzing an extensive sample providing an unprecedented level of geographic coverage. As part of the Pan African Programme: The Cultured Chimpanzee (PanAf)^38^, over an 8-year period, we non-invasively collected and genotyped >5000 wild chimpanzee faecal samples from 55 localities in 18 countries across the entire species range. We scored individual genotypes (allele length polymorphism) at up to 14 microsatellite markers, as these are cost effective and allow for accurate genotyping of non-invasively collected samples^39^. Previous studies in other species (fruit flies^40^, fish^41,42^, birds^43^, amphibians^44^, wild boars^34^, felids^45^ and beetles^46^) have demonstrated that 6–14 microsatellite loci are informative enough to even detect subtle population structure. Furthermore, a previous study of how sampling affects detection of chimpanzee population structure found that eleven loci were sufficient to overcome false signals of population structure caused by sampling bias and uncover a signal of IBD^9^. As microsatellite markers evolve rapidly and are expected to be selectively neutral, they are highly sensitive to patterns of gene flow, especially within shallow evolutionary timescales. For our analyses, we modeled our georeferenced genetic data in space by employing spatially explicit approaches to inform the distribution, thereby avoiding a priori definitions of the population structure or assumptions of homogeneous sampling, thus minimizing biases associated with previous studies.

## Results

The PanAf team and collaborators collected 5397 chimpanzee faecal samples from 55 temporary or long-term research sites spanning the geographic range of chimpanzees from 2010 to 2018 (Fig. 1). After DNA extraction and microsatellite amplification, 2497 extracts were successfully typed at up to 14 loci (range: 7–14, mean = 11.3, Supplementary Tables 1.1–1.4) and represent 939 unique individuals from 48 sampling locations (see Supplementary Note 1: ‘Genotype reconstruction’). We found that including individuals typed at seven loci (~8% of all samples) in all analyses did not affect the observed patterns of genetic differentiation compared to limiting the minimum to eight or nine loci, and this inclusion allowed for the addition of several key sampling locations in comparisons of population diversity levels. Reducing the minimum to six loci (*n* = 69) noticeably reduced differentiation across analyses.

### Influence of geographic distance on genetic distance

In an idealized model with uniform population density and dispersal, genetic differentiation among local demes is expected to increase with geographic distance (*D*). Rousset^47^ showed that, in a linear one-dimensional (1d) space, *F*_ST_/(1 – *F*_ST_) ~ *D* and *F*_ST_/(1 – *F*_ST_) ~ log(*D*) in an infinite two-dimensional (2d) space, when mutation is negligible. In principle, one may statistically fit these equations to actual data and interpret deviations from fits as signals of non-uniform spatial patterns of density and gene flow.

Actual geographical spaces are neither 1d nor infinite 2d spaces, and populations inhabiting an elongated 2d space are expected to show relationships of *F*_ST_ and *D* somewhat in-between linear and logarithmic^47^. In addition, mutation is often not negligible. Microsatellite DNA markers, in particular, have a typically fast mutation rate, and the number of repeats at a given microsatellite marker tends to change according to a stepwise mutation model (SMM)^48^, which leads to an overestimation of diversity. While a strict SMM lends itself to unbiased estimation of demographic parameters from microsatellite variation (e.g., Goldstein^49^), it rarely accurately describes the actual mutation process at microsatellite loci. Constraints to the maximum and minimum size number of repeats at a given marker^50^ are especially important, as they determine an upper limit to the genetic differentiation among pairs of populations. With these limitations in mind, modeling genetic differentiation as a function of geographical distance is still a powerful tool to identify discontinuities in the spatial structure of biological populations.

In a global, unstratified test of our data (*G* ~ *D*) in which subspecies were not defined, we found that *D* explained >30% (adjusted *r*^2^ = 0.314) of *G*, and as much as 58% (adjusted *r*^2^ = 0.579), when three obvious outlier sites were removed (Mt. Sangbé (Côte d’Ivoire), Gashaka (Nigeria) and Issa (Tanzania)). These sites were consistent outliers in all comparisons and analyses (Supplementary Fig. 3b and Supplementary Fig. 4a, b). A log-transformed model (*G* ~ log(*D*)) gave very similar results (adjusted *r*^2^ = 0.320 and 0.579 for the global model and for the model excluding outliers, respectively), as it could be expected given that chimpanzee range’s shape is fairly elongated and somewhat intermediate between a 1d and a 2d space.

**Fig. 2:**
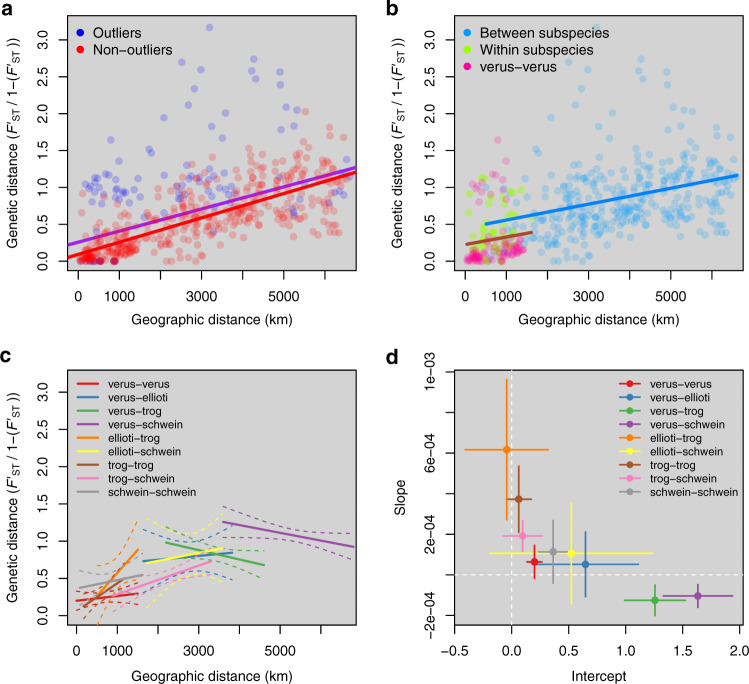
Linear regressions of genetic distance as a function of geographic distance. **a** Linear regressions of genetic distance as a function of geographic distance for all sites with at least six genotyped individuals. Blue dots represent pairwise comparisons involving outlier sites (Mt. Sangbé, Gashaka and Issa, see text). The purple line is fitted to the entire dataset, while the red line is fitted to the dataset excluding the three outlier sampling locations. When excluding the three outlier sites, geographic distance explains 58% of the genetic distance. **b** Linear regressions of genetic distance as a function of geographic distance for between- and within-subspecies comparisons. Blue dots represent between-subspecies pairwise comparisons with linear regression (blue line). Green and pink dots represent within-subspecies pairwise comparisons with linear regression (brown line) characterized by a noticeably lower *y*-intercept. *Pan troglodytes verus–P. t. verus* comparisons (pink dots), which have the lowest genetic diversity of all subspecies, make up 60% of all the within-subspecies comparisons and are largely driving this observed pattern, explaining the source of the stratification in **a**. **c** Linear regressions of genetic distance as a function of geographic distance for each subspecies comparison pair. Solid lines represent fitted regressions, and dashed lines enclose 95% confidence intervals. **d** Estimates (circles) and standard errors (crosses) of intercept and slope for each of the regressions in **c**.

Full-range data were then stratified according to whether the two sites in each pair belonged to the same or different subspecies (Fig. 2b). However, further analyses in which each subspecies pairs were separately considered (e.g., *P. t. verus–P. t. verus, P. t. verus*–*P. t. troglodytes*, etc., Fig. 2c, d) revealed that this pattern was largely driven by the overrepresentation of site pairs within *P. t. verus*, which is known to have the lowest diversity of all subspecies^15,33^, resulting in a very low intercept. Fitting *G* *~* *D* regressions within each subspecies pair, instead, did not reveal clear evidence of abrupt discontinuities across the chimpanzee range (Fig. 2c, d), but rather highlighted ‘locally’ different patterns. In particular, *P. t. verus* populations displayed low slope and intercept, which, combined with their low genetic diversity, is consistent with a relatively recent and fast demographic and spatial expansion, while *P. t. troglodytes* had low intercept but a higher slope (consistent with a more stable pattern of IBD) and there was no clear IBD pattern within *P. t. schweinfurthii* (where *G* tends to be high, irrespective of geographic distance, which can be expected given the rugged landscape in the great rift area, where three sampled sites are located). Notably, between-subspecies regressions involving *P. t. verus* (the most geographically isolated and genetically divergent subspecies^15^) were essentially flat, suggestive of saturated genetic differentiation at our markers, while the *P. t. troglodytes*–*P. t. schweinfurthii* regression was highly similar to within-subspecies regressions, with coefficients in-between *P. t. troglodytes* and *P. t. schweinfurthii* in the linear regression and almost identical to within *P. t. troglodytes* in the log-transformed regression, Fig. 2c, d.

### Historical estimated effective migration rates

To visualize chimpanzee migration patterns and test for spatial genetic structure, we employed the spatially explicit analysis implemented in the Estimated Effective Migration Surfaces (EEMS) software^51^. This analysis combines spatial and genetic data and relies on MCMC to estimate relative effective migration rates (*m*; between-deme genetic dissimilarity measured against geographic distance) modeled on a 2d surface, using a mean-centred relative scale. In this model, a ‘flat’ uniform migration surface would indicate perfect IBD across the considered range, and non-uniformity of *m* in space is interpreted as increased or decreased genetic connectivity. Besides plotting estimated migration surfaces, EEMS results differentiate areas where the probability of *m* differing from the mean rate is statistically significant (i.e., local *m* > mean *m* in >95% of the MCMC samples). Importantly, the EEMS model assumes that microsatellite markers mutate according to a strict SMM and violations of this assumption may lead to an overestimation of effective migration rates, while homoplasy caused by allelic size saturation may lead to an underestimation. Despite having some deviations from strict SMM and the presence of homoplasy that are apparent in our data, we found the EEMS model to be a good fit to our chimpanzee dataset (adjusted *r*^2^ = 0.381, Supplementary Fig. 7a). However, the observed genetic dissimilarity tended to be less than predicted by the model for very large distances, i.e., mostly involving *P. t. verus* vs. *P. t. schweinfurthii* pairs, and in particular, *P. t. verus*–Issa comparisons (Supplementary Fig. 7b), similar to our observations in the regression analyses.

**Fig. 3:**
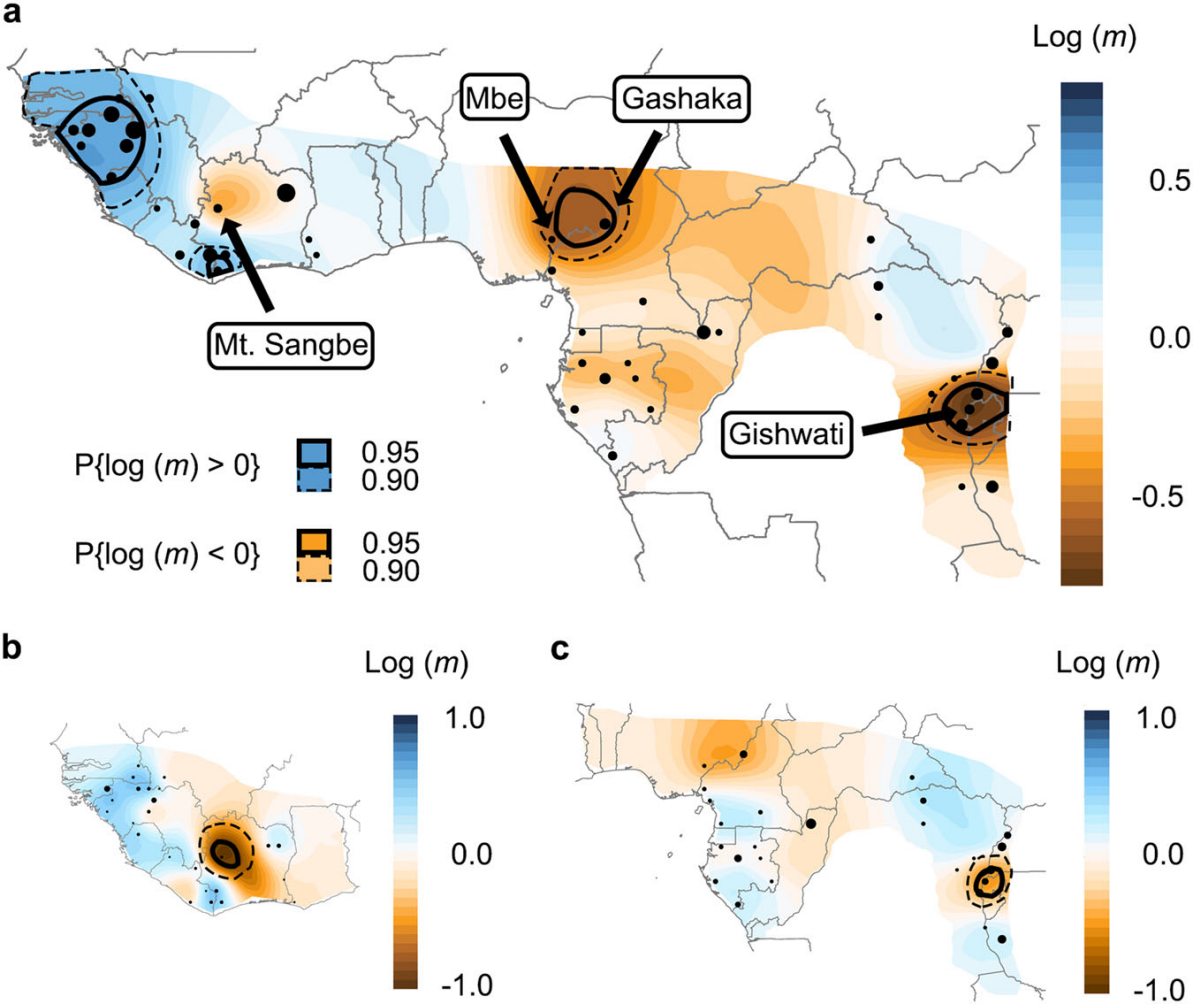
Estimated effective migration surfaces (EEMS) at different population scales. **a** Map of EEMS for the entire species. Estimated effective migration rates (*m*) are mean centred on a log_10_ scale. A value of 1 equates to a tenfold rate increase over population average, ranging from areas of low (brown) to high (blue) *m*. The intensity of the colours represents the relative difference from the population mean rates. Point diameter is proportional to the number of individuals sampled in a given deme (range = 1–72). Solid black lines indicate areas where the posterior probability of *m* differing from the mean rate is >95 percent and the dashed lines highlight areas that are >90 percent. These can be interpreted as significant effective ‘barriers’ to migration in brown areas and significant effective ‘corridors’ for migration in the blue areas. Two significant effective barriers were present: one corresponding to Gashaka and Mbe (Nigeria) and another originating from Gishwati (Rwanda) and shared with its nearest neighbours. These are localized areas of high differentiation, and when we excluded Mbe and Gishwati from the dataset, these barriers were no longer significant (Extended Data Fig. 6a, c). Notably, historical effective barriers separating the subspecies’ ranges were not detected. Effective migration rates within much of *Pan troglodytes verus* were significantly higher than average. **b** Historical EEMS map of *P. t. verus*. There was a significant barrier associated with Mt. Sangbé in Côte d’Ivoire. When we removed Mt. Sangbé the barrier was no longer significant, but it was still present, suggesting the possibility of reduced historical gene flow across Côte d’Ivoire. **c** Historical EEMS map of *P. t. ellioti*, *P. t. troglodytes* and *P. t. schweinfurthii* (collectively *ETS)*. As in **a**, removing Gishwati resulted in the barrier no longer being significant (Extended data Fig. 6c). Rates of *m* between the panels are relative and not directly comparable.

Although we observed spatial variation in *m*, we did not detect any significant signal of genetic discontinuity, or ‘barriers’ between-subspecies populations (Fig. 3a). Consistent with the divergent results from the *G* ~ *D* regression models (Fig. 2a–d), *m* was generally higher in the *P. t. verus* range than in the rest of the species range (*P. t. ellioti, P. t. troglodytes* and *P. t. schweinfurthii*, hereafter, collectively, *ETS*) and two patches of significantly high *m* were identified in the most densely sampled areas of the *P. t. verus* range. In contrast, significantly low migration was inferred for the region surrounding the Gashaka sampling location within the range of *P. t. elliotti* and in the mountainous regions of southern Uganda and Rwanda centered around Gishwati in the *P. t. schweinfurthii* range.

Since EEMS estimates relative *m* and the considerable contrast between *P. t. verus* and *ETS* may subdue more local patterns, we also performed separated analyses for *P. t. verus* and for the remaining *ETS* populations (Fig. 3b, c). The *P. t. verus* analysis (Fig. 3b) highlighted significantly low *m* associated with the outlier location of Mt. Sangbé, while the *ETS* analysis confirmed significantly lower gene flow in the mountainous region in the southeastern portion of the *P. t. schweinfurthii* range relative to the average *ETS* migration landscape. To determine whether local effects drove these effective barriers, we removed Mbe, Mt. Sangbé and Gishwati, and re-analyzed the data; all of the barriers were no longer significant (Supplementary Note 2: ‘Spatially explicit analyses (EEMS)’ and Supplementary Fig. 8a–c), indicating that these observed genetic discontinuities stemmed from local effects, and are associated with sites that are highly differentiated from surrounding locations. Importantly, even when outliers were removed, no significant genetic discontinuities were associated with boundaries between-subspecies ranges.

At the population level with *ETS* excluded, *m* in *P. t. verus* were surprisingly uniform (with the exception of Mt. Sangbé), given the high degree of topological, climate and habitat variation in West Africa (Fig. 3b). Meanwhile, *ETS* displayed a non-uniform effective migration surface, though, apart from the local barrier associated with Gishwati, no areas of significant variation in *m* were detected (Fig. 3c). A barrier to gene flow was not detected in the eastern extreme of the *ETS* range across the Albertine Rift, a region consisting of rivers, great lakes, gorges, and steep gradient valleys and mountain ranges—a landscape expected to have increased resistance to migration^52^. Conversely, latitudinal gene flow in populations east of the rift appeared to be discontinuous, where the mountainous areas of southern Uganda and Rwanda may restrict dispersal.

### Diversity rates

**Fig. 4:**
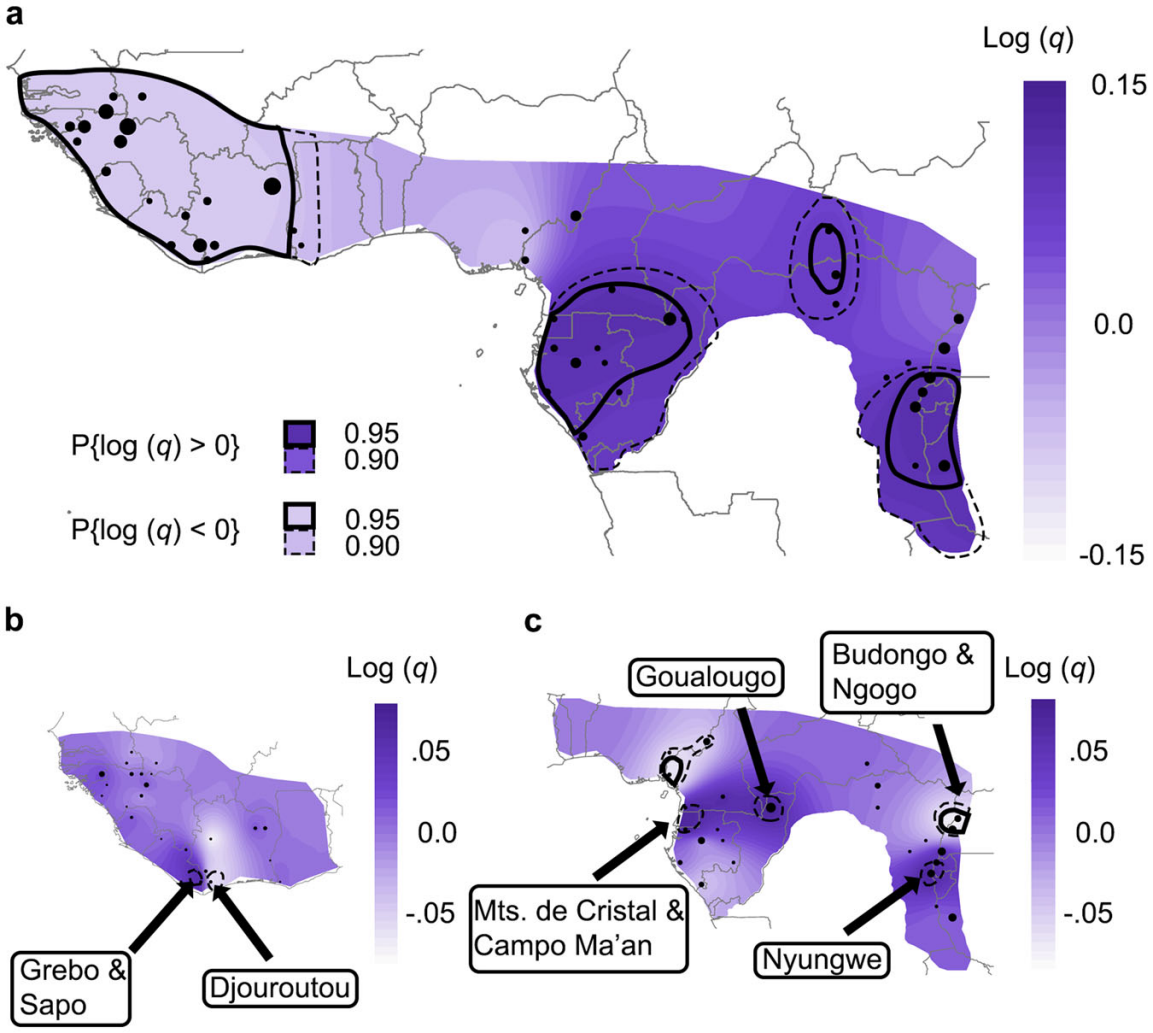
EEMS diversity rates in chimpanzees at different population scales. **a** Map of diversity rates (*q*) for the entire species. Diversity rates are mean centred (population average) on a log_10_ scale, whereby a value of 1 equates to a tenfold rate increase over population mean rate. Point diameter is proportional to the number of individuals sampled in a given deme (range = 1–72). Solid black lines indicate areas where the posterior probability of *q* differing from the mean rate is >95 percent and the dash lines highlight areas that are >90 percent. Lighter areas indicate populations where *q* is lower than the mean rate, and darker areas indicate populations where *q* is higher. Diversity rates were significantly lower in *Pan troglodytes verus*, while many of the populations in the *P. t. troglodytes* and *P. t. schweinfurthii* ranges had significantly higher rates. **b** Map of *q* in *P. t. verus*. Grebo and Sapo (Liberia) had significantly higher diversity rates than the average rate in *P. t. verus* and Djouroutou (Côte d’Ivoire) had significantly lower rates. Diversity at Mt. Sangbé (Côte d’Ivoire, white area) is also notably low, but not significant. **c** Map of *q* in *P. t. ellioti*, *P. t. troglodytes* and *P. t. schweinfurthii* (collectively *ETS*). Several sites differed significantly, with *P. t. ellioti* sites in particular displaying lower diversity rates, but overall *q* were homogeneous. Rates of *q* between panels are relative and not directly comparable.

We visualized spatial patterns of chimpanzee genetic diversity by plotting estimated relative diversity rates (*q*; pairwise within-deme genetic dissimilarity between individuals) from EEMS. At full scale, *q* in *P. t. verus* populations were significantly lower than species average (Fig. 4a). We calculated mean *q* from the EEMS output for both *P. t. verus* and *ETS*, and found that *ETS* was 1.7-fold higher, in agreement with previously published reports^15,33^. When analyzed alone (Fig. 4b), the overall distribution of diversity in *P. t. verus* was generally homogeneous, with the exception of very local significant differences in parts of Liberia (Sapo and Grebo) and Côte d’Ivoire (Djouroutou). A focused analysis of *ETS* (Fig. 4c) revealed that diversity rates tended to be higher in populations closest to the equator, and areas where rates diverged significantly were generally localized between two neighbouring populations. Interestingly, although *m* was low in the southeastern *ETS* range, diversity appeared to be relatively high. All of the populations sampled within the *P. t. ellioti* range had low relative *q*, with two sites being significantly lower than the average *ETS*. Two sampling locations in western Uganda (Budongo and Ngogo) in the *P. t. schweinfurthii* range also displayed significantly lower *q*.

## Discussion

Our results show an essentially clinal pattern of genetic variation, largely predicted by geographic distance (IBD), with strong local effects driving differentiation in a few highly isolated populations. Importantly, we did not find evidence of major discontinuities conforming to the current taxonomic subdivision. However, although effective barriers were not evident in our data, the *P. t. verus* population appears to have had a divergent history, as they display much lower genetic diversity in tests of same-subspecies among-site differentiation, while also sharing widespread higher effective migration rates across the subspecies in the whole-species EEMS analysis. Importantly, our observations of high effective migration rates in *P. t. verus* may simply be a consequence of the overall paucity of diversity in this subspecies. The EEMS model utilizes variation in genetic dissimilarity against geographic distance to estimate effective migration, therefore, high levels of genetic similarity (low diversity) in a geographically clustered subpopulation will appear to have a relatively high migration rate relative to the rest of the sampled populations. Therefore, our observation of relatively high effective migration throughout *P. t. verus* may indicate higher levels of connectivity in western chimpanzees, but may alternatively or additionally be a signal of a relatively recent (likely northward) population expansion. Indeed, much of the *P. t. verus* range is savannah mosaic habitat with discontinuous gallery forests^53^, where chimpanzees tend to have both (1) larger home ranges (over 63 km^2^)^8^ than forest-dwelling populations, to seemingly compensate for lower fruit tree density and suitable habitat availability, and (2) a concomitant increase in dispersal distances^54,55^, thereby possibly driving a signal of increased migration. However, effective migration was uniform across most of the *P. t. verus* range, as similar rates were also detected among the forest-dwelling populations in southern Liberia and western Côte d’Ivoire, where home ranges have been reported to be between 6 and 37 km^2^ in size^54^, suggesting that dynamics other than ecology are involved. This and, especially, the low diversity in *P. t. verus*, suggest that the high effective migration we observed in this subspecies should be interpreted as evidence of a population bottleneck followed by a recent and fast range expansion. Indeed, this interpretation is corroborated by among-site comparisons of mean standard deviation of allele sizes (SD; large allele-size variation) and Garza Williamson’s *M* (Supplementary Figs. 5a–d and 6a–d, and see Supplementary Note 2: ‘Detection of a population bottleneck in P. t. verus’ for detailed explanation), whereby the low SD:*M* ratio observed in *P. t. verus* suggests a loss of alleles and allele-size ranges, which can be expected for populations that have maintained a relatively small population size for a substantial number of generations (Supplementary Fig. 6a–d)^56^_._ A similar pattern was also observed in most *P. t. ellioti* samples and in the population in the Budongo/Ngogo region of Uganda, while Gashaka has both low SD and moderate *M*, indicating small long-term population size, possibly, a recent bottleneck. Conversely, populations that maintain a comparatively high SD, but display a low value of *M* in the central and eastern subspecies (e.g., Gishwati and Issa), are suggested to have experienced a more recent bottleneck (Supplementary Fig. 6a–d).

Our estimates of effective migration rates did not detect significant discontinuities corresponding with the Dahomey Gap, the Sanaga or Ubangi Rivers, the three proposed subspecies-delimiting barriers. Although our sampling scheme endeavoured to collect data from as many localities as was practicable, unavoidable sampling gaps occurred in the *P. t. ellioti* range, between *P. t. ellioti* and *P. t. troglodytes*, and in northwestern DRC between *P. t. troglodytes* and *P. t. schweinfurthii*. However, these gaps were of moderate extension and spatial homogeneity in sampling is not an assumption of either the EEMS analysis or our IBD regression, so that it is extremely unlikely that sampling gaps represent a main influence on our results. Our analyses, therefore, indicate that previous observations^11–14^ of recent genetic discontinuities in chimpanzees likely resulted, at the minimum, from the use of spatially agnostic methods that were biased by geographically clustered genetic samples. Notably, these results do not at all suggest an overall lack of genetic differentiation among chimpanzee populations set apart by geography. Though we found that genetic variation follows a generally clinal pattern across the species, we want to stress that we were specifically focused on patterns occurring on a recent timescale, i.e., within the current interglacial period. Populations are spatially and temporally dynamic, and it is likely that the range of suitable habitat for chimpanzees has fluctuated with the glacial–interglacial cycles, causing repeated population contractions and subsequent expansions^57,58^. It is important to remark that our analyses utilized microsatellite markers, which, mainly due to both their high mutation rates and polymorphism, can be very effective at revealing subtle genetic structure. However, fast mutation rate, coupled with constraints on maximum and minimum allele size, restrict the information that microsatellites may provide about deeper demographic events, leading to a shallow effective timescale. In particular, a deep divergence between populations, e.g., chimpanzee subspecies, may appear relatively shallow if there are heavy constraints on allele size (which causes differentiation to become asymptotic). However, if previously isolated populations came into contact in the more recent past, demes that are closer to the interface will exchange more migrants, and thus more alleles. The admixed alleles would diffuse along a gradient, since they would become more dispersed as distance from the interface increases. In such cases, recent gene flow and the relative underestimation of deep divergences by microsatellites may, in principle, create a smooth pattern of IBD, whereas other markers might otherwise reveal steeper gradients. This makes microsatellites highly effective for assessing recent patterns of population structure, as signals of ancient contraction–expansion events are flattened by allele size constraints and relatively high mutation rates. Finally, we wish to stress that gradients are not expected to occur by chance alone. Therefore, observations of IBD in our data represent, at least, clear evidence of recent connectivity among populations.

The chimpanzee populations that appear considerably more differentiated than expected by IBD alone, e.g., Mt. Sangbé, Gashaka and Issa, are known to have experienced isolation and decline in recent generations as a consequence of local anthropogenic pressures^59–62^. Therefore, their observed differentiation most likely stems from loss of alleles due to random drift in extremely small populations.

Our data suggest that, much like humans, chimpanzees likely experienced periods of substantial genetic connectivity across a geographic range encompassing a broad range of habitats, a finding that may point to important evolutionary implications. Environmental heterogeneity and population size act synergistically to influence rates of adaptive evolution. Advantageous mutations are more likely to become fixed in small populations occupying uniform habitats, and are less likely to become fixed in a large population occupying a diverse habitat^2^. Therefore, as humans and chimpanzees are both widely distributed across a variety of habitat types, evolutionary adaptation is expected to be slow in these species, especially given their long life histories. Hominins have long employed behavioural flexibility to mitigate biological limitations, exploit new resources^5^, inhabit challenging environments (i.e., use of fire) and migrate out of Africa on several occasions before modern times^4,5^. Since our results have demonstrated that recent genetic variation in chimpanzees contains little geographic structure on a broad scale, behavioural flexibility may also play an important evolutionary role in their adaptability to environmental heterogeneity^63^. Chimpanzees have diverse and variable behavioural repertoires^64,65^ that often vary among nearby communities^66^, they utilize learned techniques to harvest otherwise unobtainable foods^67^ and their bevavioural diversity increases with environmental variability^68^. Indeed, chimpanzees living in arid habitats employ specialized behaviours to regulate the thermal exposure^8^ and spontaneously innovate novel behaviours in response to increased environmental complexity arising from anthropogenic pressures^69–71^.

Although our data point to diffuse genetic connectivity in chimpanzees, this signal originates from recent historical patterns in previous generations and does not represent the current potential for gene flow. Indeed, we found that several local chimpanzee populations are very strongly isolated from a genetic perspective, and many more are known to have become fragmented and/or have undergone precipitous declines in recent decades due to anthropogenic factors, such as habitat fragmentation, hunting and disease transmission^72–74^. *P. t. schweinfurthii*, *P. t. troglodytes* and *P. t. ellioti* are recognized as endangered^19^, while *P. t. verus* is recognized as critically endangered^73^. Though our results indicate a high level of recent genetic connectivity in chimpanzees, we do not suggest that the distinction among the subspecies populations is irrelevant. Each subspecies range hosts populations that contribute unique behaviours to the species as a whole, and our findings suggest that genetic diversity in chimpanzees is distributed throughout the taxon, underscoring the need to maintain regionally driven conservation approaches. However, these results also highlight the need to preserve and restore corridors to facilitate connectivity among remaining populations to avoid inbreeding depression and the accumulation of deleterious alleles that negatively affect fitness. All populations possess unique traits that cumulatively reflect the full genetic and behavioural diversity observed in chimpanzees. Continued isolation and further population decline will irrevocably erode the overall viability of this critical keystone species, ultimately imperiling their long-term fitness. If connectivity between populations is not restored or deteriorates, the geographic distribution we observe in chimpanzees today will shape the genetic structure of future generations^75^, which may have negative evolutionary consequences^2^, and ultimately jeopardizes the long-term survival of wild populations.

## Methods

### Sample collection and preparation

As part of the PanAf, 5397 chimpanzee faecal samples were non-invasively collected at 55 temporary or long-term research sites across the species range (Fig. 1, Supplementary Table 1.3 and Supplementary Fig. 3c), preserved according to the two-step ethanol–silica method^76^ and stored in the field for up to 2 years. Upon arrival to the lab, samples were stored at −20 °C. DNA extracts were isolated either manually, using the QIAamp Stool Kit (Qiagen), or using an automated process employing the QIAamp 96 PowerFecal QIAcube HT robot (Qiagen), per manufacturer instructions, modified by incorporating a pre-treatment step to improve the DNA quality and yield (Supplementary Note 1: ‘Laboratory methods’). Fourteen unlinked microsatellite loci and one sex-determining locus (amelogenin) were amplified using a two-step multiplex process^77^ with slight modifications (Supplementary Note 1: ‘Laboratory methods’ and Supplementary Fig. 1). PCR products were analyzed using an ABI Prism 3730 genetic analyzer (Thermo Fisher Scientific) and allele sizes were measured relative to ROX labeled HD400 internal size standard using Genemapper version 5.0 (Thermo Fisher Scientific). Homozygotes were identified by three identical PCR replicates and heterozygotes were confirmed by at least two unambiguous PCR replicates of each allele^77^ (Supplementary Note 1: ‘Genotype reconstruction’).

### Analyses

Cervus 3.0.7 (ref. ^78^) was used to calculate allele frequencies, determine the minimum number of loci needed to discriminate individuals (P_IDsib_)^79^, and to assess the degree of relatedness of individuals within sites (Supplementary Note 1: ‘Genotype reconstruction’ and Supplementary Table 5). Based on the allele frequency data, we determined that we needed eight loci to be 99.9 percent certain that two identical genotypes originated from the same individual rather than full siblings^79^. In cases where genotypes were unique (not matching to any other genotype), individuals were typed at a minimum of seven loci.

We employed two strategies to assess genetic continuity at the species, subspecies and site levels: (1) we examined the relationship between genetic and geographic distances by applying simple linear regression functions to multiple levels of our data, and (2) we used a spatially explicit analysis that identifies significant spatial deviations from clinal variation (genetic discontinuity). We also performed Mantel, partial Mantel and STRUCTURE analyses on the dataset, as they are standard practice in population structure studies; however, reliable inferences could not be drawn as results were severely confounded by biases in the data. Instead, we provide in-depth details about the modeling and results of these analyses in Supplementary Note 2: ‘Cluster (STRUCTURE) analysis and stratified and partial Mantel tests’ (Supplementary Fig. 2a–c and Supplementary Table 2.1).

We utilized linear regression analyses (*G* ~ *D*) to assess the relationship between genetic distance (*F*′_ST_/(1 − *F*′_ST_))^80^ and geographic distance (least cost paths; LCPs) at the species, between and within-subspecies and subspecies-comparisons levels. For our analyses, we calculated *F*′_ST_ as a suitable estimator of *F*_ST_, which adjusts for differing levels of genetic diversity arising from variation in effective population sizes (*N*_e_) among sampling locations and mutation rate among loci, while not being dependent on within-site diversity^80^. *F*′_ST_/(1 − *F*′_ST_) (hereafter *G*) was modeled as a function of LCP estimators of *D*, which calculate pairwise distance between sampling localities based on the presence of suitable chimpanzee habitat spanning back 120 ky BP^57^, and include topological features that could cause resistance to gene flow. We used LCPs specifically to force distance measurements around the presence of large water bodies, such as the Atlantic Ocean, where chimpanzee migration is not possible (Supplementary Fig. 3a, c).

To test for effective barriers to migration and to plot the distribution of chimpanzee genetic diversity across their range, we used the spatially explicit EEMS program^51^. EEMS estimates historical migration rates that would have given rise to the observed spatial distribution of genetic diversity in the data under idealized conditions, i.e., random mating, neutral selection and constant population size. This method uses georeferenced genetic data to assess diversity in a spatial context; therefore, in contrast to Bayesian clustering algorithms, it does not require sampling homogeneity, ideal for our unbalanced, heterogeneous dataset. Furthermore, the EEMS analysis is not biased by the presence of IBD in the data, rather, decreasing genetic similarity as a function of distance is the null assumption of the model. EEMS generates maps that display mean-centred relative patterns of genetic diversity (*q*) and relative effective migration (*m*) rates, therefore, high levels of genetic differentiation appear as divergent from these means.

In a study of both African elephant species Petkova et al.^51^, demonstrated that a single highly polymorphic locus was able to replicate qualitatively similar effective migration surfaces as when using their entire panel of loci. More importantly, this locus was able to correctly identify the barrier and differentiation between two geographically proximate and closely related species, which is particularly notable as they are known to hybridize where their respective ranges overlap^81^. In a study of three closely related sympatric beetle species that inhabit forested areas surrounding Kyoto, Japan, the authors used EEMS to examine the spatial distribution of genetic diversity in the presence of habitat discontinuity^46^. Using a range of nine to ten loci, they detected discrete spatial structuring in two out of the three species, which directly correlated with differing levels of dispersal capability and host species specificity. These results suggest that our use of 14 loci was sufficient for detecting the presence of genetic structure both within subpopulations and between closely related species, especially given the geographic range and depth of sampling in the present study.

We found that the magnitude of differentiation between *P. t. verus* and *ETS* populations saturated the signal of regional patterns, so it was necessary to perform analyses of these two populations independently. We found several barriers to *m* that appeared to be associated with individual populations so we performed additional analyses excluding these (see Supplementary Note 2: ‘Spatially explicit analyses (EEMS)’ and Supplementary Fig. 8a–c). The genetic data analyzed in this study retain the history of hundreds of generations or more, therefore, excluding potential areas of past occupation and migration corridors will bias the results. Since the EEMS model identifies areas where genetic differentiation is higher or lower than expected (IBD), areas where gene flow is restricted or non-existent will be inferred as a significant barrier. Thus, expanding the boundaries of the analysis is necessary and will not lead to erroneous conclusions. The historical range of chimpanzees has likely expanded and contracted over time^7^, so we accordingly extended the current northern boundaries of the species range by 300 km in our population grids for EEMS. We also included the Dahomey Gap as a functional part of the chimpanzee range, as it has fluctuated in size and featured rainforest as recently as 4 ka BP, a period within the timescale of this study^21^. For grid density, we performed iterative analyses to ascertain the optimal number of demes that provided the best model fit for analyses at the species (800 demes), *P. t. verus* (300 demes) and *ETS* (490 demes) population levels^51^. For all analyses, to ensure the models reached stability, three independent MCMC chains were run preceded by 500,000 steps of burn-in, at lengths of 6,000,000 repetitions, each beginning with different random seeds.

### Statistics and reproducibility

We performed all analyses from a pool of 939 individual genotypes originating from 48 sampling locations with a minimum inter-site distance of 41 km (mean pairwise distance 2843 km; estimated from LCPs). When necessary, we balanced our dataset by imposing a lower limit of 6 and an upper limit of 20 (using a randomized drawing process) on the number of genotypes originating from the same sampling location (see Supplementary Note 2: ‘Isolation by distance (IBD) and distance estimators’). For the EEMS analyses, since balancing was unnecessary, all samples for which geospatial data were recorded (932) were used in the species-wide scale and were divided for focused analyses of *P. t. verus* (522) and *ETS* (410). Detailed descriptions of all analyses and *p* values are provided in the text and Supplementary Note 2: ‘Population genetics analyses’.

### Reporting summary

Further information on research design is available in the Nature Research Reporting Summary linked to this article.

## Supplementary information

Supplementary Information

Reporting Summary

## Data Availability

The data from the present study are available from the corresponding authors upon reasonable request.
