# Supplementary material for: Recent genetic connectivity and clinal variation in chimpanzees
Source: Commun Biol. 2021 Mar 5;4:283. doi: 10.1038/s42003-021-01806-x (PMC7935964; doi:10.1038/s42003-021-01806-x)
Supplement: Supplementary file 1 — Supplementary Information [file 42003_2021_1806_MOESM1_ESM.pdf]

Recent genetic connectivity and clinal variation in chimpanzees

Jack D. Lester[1*], Linda Vigilant[1], Paolo Gratton[1], Maureen S. McCarthy[1], Christopher D. Barratt[2], Paula Dieguez[1], Anthony Agbor[1], Paula Álvarez-Varona[3], Samuel Angedakin[1], Emmanuel Ayuk Ayimisin[1], Emma Bailey[1], Mattia Bessone[1], Gregory Brazzola[1], Rebecca Chancellor[4], Heather Cohen[1], Emmanuel Danquah[5], Tobias Deschner[1], Villard Ebot Egbe[1], Manasseh Eno-Nku[6], Annemarie Goedmakers[7], Anne-Céline Granjon[1], Josephine Head[1], Daniela Hedwig[8], R. Adriana Hernandez-Aguilar[3,9], Kathryn J. Jeffery[10], Sorrel Jones[1], Jessica Junker[1], Parag Kadam[11], Michael Kaiser[1], Ammie K. Kalan[1], Laura Kehoe[12], Ivonne Kienast[1], Kevin E. Langergraber[13], Juan Lapuente[1,14], Anne Laudisoit[15,16], Kevin Lee[1], Sergio Marrocoli[1], Vianet Mihindou[17,18], David Morgan[19], Geoffrey Muhanguzi[20], Emily Neil[1], Sonia Nicholl[1], Christopher Orbell[21], Lucy Jayne Ormsby[1], Liliana Pacheco[3], Alex Piel[22], Martha M. Robbins[1], Aaron Rundus[23], Crickette Sanz[24,25], Lilah Sciaky[1], Alhaji M. Siaka[26], Veronika Städele[1], Fiona Stewart[27], Nikki Tagg[28], Els Ton[7], Joost van Schijndel[7], Magloire Ka+mbale Vyalengerera[1], Erin G. Wessling[29], Jacob Willie[28], Roman M. Wittig[1,30], Yisa Ginath Yuh[1], Kyle Yurkiw[1,31], Klaus Zuberbuehler[20,32,33], Christophe Boesch[1], Hjalmar S. Kühl[1,2] & Mimi Arandjelovic[1*]

*corresponding authors

---

[1]Max Planck Institute for Evolutionary Anthropology (MPI EVAN), Deutscher Platz 6, 04103 Leipzig
[2]German Centre for Integrative Biodiversity Research (iDiv) Halle-Jena-Leipzig, Leipzig, Germany
[3] Jane Goodall Institute Spain and Senegal, Dindefelo Biological Station, Dindefelo, Kedougou, Senegal
[4]West Chester University, Depts of Anthropology & Sociology and Psychology, West Chester, PA, 19382 USA
[5]Department of Wildlife and Range Management, Faculty of Renewable Natural Resources, Kwame Nkrumah University of Science and Technology, Kumasi, Ghana
[6]WWF Cameroon Country Programme Office, BP6776; Yaoundé, Cameroon
[7]Chimbo Foundation, Amstel 49, 1011 PW Amsterdam, Netherlands
[8]Elephant Listening Project, Center for Conservation Bioacoustics, Cornell Lab of Ornithology, Cornell University, 159 Sapsucker Woods Road, Ithaca, NY 14850, USA
[9]Department of Social Psychology and Quantitative Psychology, Faculty of Psychology, University of Barcelona, Passeig de la Vall d'Hebron 171, 08035, Spain
[10]Biological and Environmental Sciences, Faculty of Natural Sciences, University of Stirling, Stirling FK9 4LA, UK
[11]University of Cambridge, Pembroke Street, Cambridge, UK CB2 3QG
[12]Wild Chimpanzee Foundation (WCF), Deutscher Platz 6, 04103 Leipzig
[13]School of Human Evolution and Social Change, Arizona State University, 900 Cady Mall, Tempe, AZ 85287 Arizona State University, PO Box 872402, Tempe, AZ 85287-2402 USA
[14]Comoé Chimpanzee Conservation Project, Kakpin, Comoé National Park, Côte d'Ivoire
[15]Ecohealth Alliance, 460 west 34th street, Ste1701, 10001 New York, USA
[16]University of Antwerp, Campus Drie Eiken, lokaal D.133, Universiteitsplein 1 - 2610 Antwerpen, Belgium
[17]Agence National des Parcs Nationaux (ANPN) Batterie 4, BP20379, Libreville, Gabon
[18]Ministère des Eaux, des Forêts, de la Mer, de l'Environnement, Chargé du Plan Climat, des Objectifs de Développement Durable et du Plan d'Affectation des Terres, Libreville, Gabon
[19]Lester E. Fisher Center for the Study and Conservation of Apes, Lincoln Park Zoo, 2001 North Clark Street, Chicago, Illinois 60614 USA
[20]Budongo Conservation Field Station, PO Box 362, Masindi, Uganda
[21]Panthera, 8 W 40TH ST, New York, NY 10018, USA
[22] Department of Anthropology, University College London, 14 Taviton St, Bloomsbury London, WC1H OBW, UK
[23]West Chester University, Department of Psychology, 700 S High St., West Chester, PA, 19382 USA
[24]Washington University in Saint Louis, Department of Anthropology, One Brookings Drive, St. Louis, MO 63130, USA
[25]Wildlife Conservation Society, Congo Program, B.P. 14537, Brazzaville, Republic of Congo
[26]National Protected Area Authority, Sierra Leone
[27]School of Biological & Environmental Sciences, Liverpool John Moores University, James Parsons Building, Byrom street, Liverpool, L3 3AF, UK

[28]KMDA, Centre for Research and Conservation, Royal Zoological Society of Antwerp, Koningin Astridplein 20-26, B-2018 Antwerp, Belgium
[29]Department of Human Evolutionary Biology, Harvard University, 11 Divinity Avenue Cambridge, MA 02138 USA
[30]Taï Chimpanzee Project, Centre Suisse de Recherches Scientifiques, Abidjan, Côte d'Ivoire
[31]Pan Verus Project Outamba-Kilimi National Park, Sierra Leone
[32]Université de Neuchâtel, Institut de Biologie, Rue Emile-Argand 11, 2000 Neuchâtel, Switzerland
[33]School of Psychology and Neuroscience, University of St Andrews, St Andrews, UK
Email: jack_lester@eva.mpg.de; arandjel@eva.mpg.de

Supplementary Information

Table of contents

- Supplementary Note 1 ………………………………………………………………… 4
    - Laboratory methods ……………………………………………………… 4
    - Genotype reconstruction …………………………………………………… 7
    - Null alleles ………………………………………………………………… 13
    - Relatedness ………………………………………………………………… 15
- Supplementary Note 2 ………………………………………………………………… 18
    - Cluster (STRUCTURE) analyses …………………………………………… 18
    - Isolation by distance (IBD) and distance estimators ……………… 30
    - Stratified and partial Mantel tests ……………………………………… 31
    - Linear regressions ……………………………………………………… 33
    - Detection of a population bottleneck in *Pan troglodytes verus* …. 37
    - Spatially explicit analyses (EEMS) ………………………………… 43
- Supplementary references ……………………………………………………… 51

Supplementary Note 1

Summary: 939 unique genotypes were generated from DNA isolated from non-invasively collected wild-chimpanzee faecal samples. The sampling range required the construction of *de novo* allele bins to accommodate the variety of alleles among loci across the entire species to capture all detectable alleles. A parentage test between all genotypes yielded an average of 1.85% first-order relatives, which falls within the expected proportion[15,16]. Null alleles were detected but are due to subpopulation structure (Wahlund effect)[13] and do not conflict with the overall results of the study.

Laboratory methods

For DNA extracts obtained using the QIAamp 96 PowerFecal QIAcube HT (Qiagen) robot, 120 μl of solution were isolated from desiccated faecal samples which varied from 10 to 80 mg starting weight according to substrate type and density. DNA isolates were purified as per manufacturer instructions, modified by incorporating the following pretreatment procedure. Faecal samples were added to 2 ml reaction tubes prepared with sterilized 5mm stainless steel beads. 650 μl of QIAGEN Lysis Solution, pre-warmed to 70° C, was added to each reaction tube and then homogenized thoroughly by vortexing on the highest setting for 10 minutes, followed by centrifugation for 3 min at 12,000 rpm. Between 200 and 550 μl supernatant was pipetted from each reaction tube into a QIAGEN S-block. We then added Add 150 μl of QIAGEN Inhibitor Removal Buffer to each reaction well of the S-block and then sealed it with adhesive film. The contents were mixed thoroughly using a vortex, followed by a 5-minute incubation at 4°C then

continuous centrifugation for 5 minutes at 2800 rpm. We next added 20 μl QIAGEN Proteinase K to each reaction well and then resealed with new adhesive film. We utilized a specialized QIAGEN QIAamp Fast DNA Stool Qiacube HT MB software protocol that was modified with a pretreatment protocol, which included a filtration step that required the use of an S-Block fitted with ports for a vacuum step, a QIAGEN Turbo filter and the addition of 250 μl of 100% ethanol to each reaction well during the procedure. These steps were necessary to accommodate the desiccated faecal samples used in this study, improve DNA yield and PCR amplification, and prevented instrument clogging during the extraction process.

DNA extracts were PCR amplified at 14 unlinked, polymorphic microsatellite loci and an additional sex-determining locus (amelogenin) using a two-step multiplex process[1,2] with the following modifications. Rather than a second step singleplex PCR, we used multiplexes of subsets of the target loci to improve workflow efficiency. In the first step we used a primer concentration of 0.15 mM as in the two-step multiplex protocol[1], but in the second step we adjusted primer concentrations for each individual primer pair to achieve consistent levels of amplification across all loci in each respective PCR group[3]. PCR thermal cycling took place in PTC-200 (MJ Research), T100 and C1000 Touch (Biorad) thermal cyclers (See Supplementary Figure 1 for PCR protocol and thermal cycling conditions).

First multiplex step

Reagent	Volume (µl) per reaction
Type-it Multiplex PCR Master Mix	10
Primer mix: Forward and reverse unlabeled (100 mM)	1.2
Nanopure $H_2$0	3.8
Template DNA	5
Total Volume	20

Second multiplex step group 1

Reagent	per reaction
Type-it Multiplex PCR Master Mix	5.0
D7s817$_{F2}$[1]	0.30
D2s1326$_{R2}$	0.30
D18s536	0.16
D11s2002$_{R2}$	0.16
Amelogenin	0.30
Nanopure $H_2$0	1.28
Diluted PCR product[2]	2.5
Total Volume	10

Second multiplex step group 2

Reagent	Volume (µl) per reaction
Type-it Multiplex PCR Master Mix	5.0
D5s1470$_{R\text{-}pigtailed}$	0.10
D1s1622	0.12
D3s2459	0.12
D9s910	0.08
D4s1627$_{R2}$	0.10
Nanopure $H_2$0	1.98
Diluted PCR product[2]	2.5
Total Volume	10

Second multiplex step group 3a[3]

Reagent	Volume (µl) per reaction
Type-it Multiplex PCR Master Mix	5.0
D1s1656	0.09
D3s3038$_{R2}$	0.3
D7s2204	0.3
D14s306	0.18
Nanopure $H_2$0	2.2
Diluted PCR product[2]	2.5
Total Volume	10

Second multiplex step group 3b

Reagent	Volume (µl) per reaction
Type-it Multiplex PCR Master Mix	5.0
D5s1457	0.08
D10s676	0.08
D12s66$_{R2}$	0.08
D6s1056	0.08
Nanopure $H_2$0	2.2
Diluted PCR product*	2.5
Total Volume	10

PCR Thermal cycling conditions (First multiplex step)

Temperature (°C)	Time (mm:ss)	
95	5:00	
94	0:20	29 Cycles
57	1:00	29 Cycles
72	0:30	29 Cycles
72	30:00	29 Cycles
10	10:00	

PCR Thermal cycling conditions (Second multiplex step group 1)

Temperature (°C)	Time (mm:ss)	
95	5:00	
94	0:20	28 Cycles
61	1:00	28 Cycles
72	0:30	28 Cycles
72	30:00	28 Cycles
10	10:00	

PCR Thermal cycling conditions (Second multiplex step group 2)

Temperature (°C)	Time (mm:ss)	
95	5:00	
94	0:20	28 Cycles
59	1:00	28 Cycles
72	0:30	28 Cycles
72	30:00	28 Cycles
10	10:00	

PCR Thermal cycling conditions (Second multiplex step group 3a)

Temperature (°C)	Time (mm:ss)	
95	5:00	
94	0:20	29 Cycles
56	1:00	29 Cycles
72	0:30	29 Cycles
72	30:00	29 Cycles
10	10:00	

PCR Thermal cycling conditions (Second multiplex step group 3b)

Temperature (°C)	Time (mm:ss)	
95	5:00	
94	0:20	29 Cycles
58	1:00	29 Cycles
72	0:30	29 Cycles
72	30:00	29 Cycles
10	10:00	

[1]Volume amounts are a combined total of both forward and nested reverse primers in equal quantities.
[2]PCR product from the first Multiplex step was diluted 1:100 with nanopure H_2O.
[3]Loci in group 3a suffered from poor amplification or low allelic diversity, consequently group 3b was utilized for most of the project.

Supplementary Figure 1. Diagram of PCR preparation and thermal-cycling conditions for first and second multiplex steps. We amplified all loci used in the study in the first step. We then amplified the PCR product (1:100 dilution) in smaller targeted groups of four or five loci, segregated by optimal annealing temperature. The combination of primers in the second multiplex step, Group 3a, did not reliably amplify alleles across loci, therefore for the second half of the study we assembled a new combination, Group3b.

Genotype reconstruction

In a reliability test[4], we determined that two replicates were required to achieve greater than 99% certainty of correctly identifying homozygotes as follows. For each locus, we summed the number of occurrences in which one of the two alleles failed to amplify in heterozygotes and divided the total by the sum of all amplifications in heterozygotes for which at least one allele amplified[1]. Nevertheless, as a conservative measure, we only confirmed homozygotes with a minimum of three identical replicates and heterozygotes were confirmed by at least two unambiguous replicates of each allele (Supplementary Table 1.1).

Supplementary Table 1.1. Allelic dropout rate and number of replicates for 99% confidence of homozygote assignment ((Allelic dropout rate)^X < 0.01. Homozygote assignment confidence was calculated by finding the value of the exponent (x) in which allelic dropout rate (allelic dropout divided by total possible amplifications) was less than 0.01 for each locus.

Locus	Allelic dropout	Total possible	Allelic dropout rate	Allelic dropout rate^2	Number of replicates (x)
D7s817$_{F2}$	410	11098	0.0369	0.00136	2
D2s1326$_{R2}$	364	8522	0.0427	0.00182	2
D18s536	337	8644	0.0390	0.00152	2
Amelogenin	414	9916	0.0418	0.00174	2
D11s2002$_{R2}$	389	9168	0.0424	0.00180	2
D5s1470$_{R\text{-}Pigtailed}$	367	8826	0.0416	0.00173	2
D1s1622	233	4496	0.0518	0.00269	2
D3s2459	412	8794	0.0469	0.00219	2
D9s910	386	11252	0.0343	0.00118	2
D4s1627$_{R2}$	369	7898	0.0467	0.00218	2
D5s1457	371	9992	0.0371	0.00138	2
D3s3038	34	944	0.0360	0.00130	2
D10s676	451	9598	0.0470	0.00221	2
D12s66	245	5812	0.0422	0.00178	2
D6s1056	297	4194	0.0708	0.00501	2

We established a set of allele bins suitable for all of our samples. Microsatellite loci have a high mutation rate[5], that tends toward asymmetry[6,7] and follow several mutation models; stepwise, infinite allele (point mutations) and two phase[3,8,9]. We began by using established alleles and bin ranges put forth by previous studies in *Pan troglodytes schweinfurthii*[2], and scored 14 loci across all 4 subspecies using these alleles. We then evaluated each locus for clustering of alleles by sorting raw allele sizes. The measured size of an allele will vary due to instrument precision and variation in measurement conditions. A previous study measuring the precision of a capillary array electrophoresis genetic analyser (ABI 3100), similar to the one we employed in our study (ABI 3730), showed the instrument measured a mean range of 0.76 base pairs (bp), with a standard deviation of 0.38 bp about the mean, across 51 alleles distributed among 7 loci[10]. Our mean range was 1.11 bp with a standard deviation of 0.56 bp (Supplementary Table 1.2).

Supplementary Table 1.2. Summary of alleles across 14 microsatellite loci. Allelic diversity and mean size range measured in base pairs (bp) per locus.

Locus	Number of alleles	Mean allele-size range (bp)	Standard deviation	Standard error
$D7s817_{F2}$	17	1.19	± 0.55	± 0.13
$D2s1326_{R2}$	32	1.13	± 0.56	± 0.10
D18s536	14	1.02	± 0.51	± 0.14
$D11s2002_{R2}$	22	1.00	± 0.49	± 0.11
$D5s1470_{R\text{-}Pigtailed}$	16	1.33	± 0.81	± 0.20
D1s1622	18	1.01	± 0.54	± 0.14
D3s2459	20	1.39	± 0.81	± 0.18
D9s910	9	1.33	± 0.38	± 0.13
$D4s1627_{R2}$	14	0.80	± 0.20	± 0.05
D5s1457	22	1.16	± 0.76	± 0.17
$D3s3038_{R2}$	12	0.78	± 0.29	± 0.09
D10s676	18	1.38	± 1.08	± 0.24
D12s66	19	1.41	± 0.62	± 0.16
D6s1056	16	0.60	± 0.24	± 0.06
Total average	17.79	1.11	± 0.56	± 0.14

Microsatellite alleles typically mutate following a stepwise model, whereby alleles mutate by an expansion or a contraction of one or more locus-specific repeat units, leading to a consistent pattern (motif). Another, less common mutation model in microsatellites, the infinite allele model, occurs when an allele changes state by a single base pair (bp), also known as an instert-deletion (indel). This leads to a disruption of the motif and can give rise to a new motif within a locus following subsequent stepwise mutation events of the original indel. When identifying allele clusters we observed numerous cases of “alleles” that fell within the gaps between repeat-unit clusters or at the extreme ends of the allele range, likely a result of an indel. If they were recorded in fewer than five individuals or seemed spurious, we tested the putative alleles by

amplifying them in a singleplex PCR as confirmation of their size and distinction from other alleles. We retained only those that unambiguously produced the same allele sizes as was recorded in the original genotypes. We noted that the single-bp indel alleles tended to cluster geographically, for which we even identified several short motifs. We also detected the presence of heterozygotes that had both a copy of a common standard allele and a copy of an indel allele, as well as homozygotes with two copies of an indel allele, further supporting their distinct identity.

We used the R package 'CongenR'[11] to assign alleles and assemble genotypes. We then analysed our genotypes using Cervus 3.0.7[12] to calculate allele frequencies, determine the minimum number of loci needed to discriminate individuals, assess null alleles, identify recaptures and to perform first-order relatives analyses. Using the resulting allele-frequency data we calculated that a minimum of eight loci were necessary to be confident that two matching genotypes were obtained from the same individual rather than a full sibling ($P_{IDsib} < 0.001$). For all unique genotypes (sampled only once), we used a minimum of seven loci for inclusion in our analyses. This resulted in a total of 939 unique individuals spanning 48 sampling locations as 7 sites did not yield any usable genotypes (Supplementary Table 1.3 and 1.4). Unfortunately, nearly every site in the *P.t. ellioti* range and around the Sanaga River yielded very few usable samples. Only sampling locations, Gashaka and Korup, satisfied the minimum number of usable genotypes (6) for estimations of F'_{ST}, somewhat limiting our inferences about *P.t. ellioti* in these analyses. It is unclear which environmental, dietary, or handling conditions may have contributed to this limitation. Due to their close proximity (less than 15km apart), Sobory and Bakoun (Guinea), Taï Ecotourism and Taï R (Côte d'Ivoire),

and Comoé, Comoé East and GEPRENAF (Côte d'Ivoire) were each considered as single sampling locations in spatially agnostic analyses and calculations of site level genetic differentiation to reduce the effect of overrepresentation of the allele frequencies from these localities. This resulted in a minimum distance between sampling locations of 41 km.

Supplementary Table 1.3. Summary of genetics results categorized by sampling location. Sampling locations are listed by subspecies from west to east, then alphabetically, by country first, then by field-site designation.

Sampling location	Country	Subspecies population	Total number of samples	Number of individuals typed at 7 or more loci	Number of individuals typed at 10 or more loci	Mean loci typed
Azagny	Côte d'Ivoire	*Pan troglodytes verus*	4	0	0	0.0
Boundiale	Côte d'Ivoire	*P.t. verus*	2	0	0	0.0
Comoé-East [2]	Côte d'Ivoire	*P.t. verus*	36	3	3	13.0
Comoé [2]	Côte d'Ivoire	*P.t. verus*	312	49	46	12.4
Djouroutou	Côte d'Ivoire	*P.t. verus*	166	27	23	11.6
GEPRENAF [2]	Côte d'Ivoire	*P.t. verus*	133	20	18	11.8
Mt. Sangbé	Côte d'Ivoire	*P.t. verus*	98	15	12	11.5
Taï-ecotourism [1]	Côte d'Ivoire	*P.t. verus*	127	10	5	9.9
Taï-R [1]	Côte d'Ivoire	*P.t. verus*	121	19	14	11.1
Ankasa	Ghana	*P.t. verus*	3	1	1	12.0
Bakoun [3]	Guinea	*P.t. verus*	147	41	37	11.9
Sangaredi	Guinea	*P.t. verus*	116	17	12	10.8
Sobory [3]	Guinea	*P.t. verus*	127	28	22	11.1
Sobeya	Guinea	*P.t. verus*	228	46	27	10.2
Boe	Guinea Bissau	*P.t. verus*	118	72	62	11.0
East Nimba	Liberia	*P.t. verus*	137	14	11	11.7
Grebo	Liberia	*P.t. verus*	200	23	18	10.9
Liberia Nationwide Survey [4]	Liberia	*P.t. verus*	37	7	4	9.6
Sapo	Liberia	*P.t. verus*	223	17	10	10.0
Bafing	Mali	*P.t. verus*	107	15	14	12.7
Dindefelo	Senegal	*P.t. verus*	184	58	57	12.6
Kayan	Senegal	*P.t. verus*	76	15	14	11.4
Loma	Sierra Leone	*P.t. verus*	14	0	0	0.0
Outamba-Kilimi	Sierra Leone	*P.t. verus*	90	24	16	10.0
Korup	Cameroon	*P.t. ellioti*	125	7	3	8.7
Mt. Cameroon	Cameroon	*P.t. ellioti*	263	4	4	12.3
Gashaka	Nigeria	*P.t. ellioti*	102	30	21	10.0
Mbe	Nigeria	*P.t. ellioti*	37	5	1	8.2
Campo Ma'an	Cameroon	*P.t. troglodytes*	74	1	0	8.0
La Belgique	Cameroon	*P.t. troglodytes*	186	8	4	9.8
Conkouati	Congo	*P.t. troglodytes*	140	17	12	10.1
Goualougo	Congo	*P.t. troglodytes*	92	49	44	12.2
Equatorial Guinea Nationwide Survey [5]	Equatorial Guinea	*P.t. troglodytes*	9	3	2	11.0
Bateke	Gabon	*P.t. troglodytes*	45	3	2	11.3
Ivindo	Gabon	*P.t. troglodytes*	2	2	1	9.5
Loango	Gabon	*P.t. troglodytes*	145	12	9	11.8
Lope	Gabon	*P.t. troglodytes*	139	29	23	11.4
Mts de Cristal	Gabon	*P.t. troglodytes*	136	9	9	13.0
Chinko	CAR	*P.t. schweinfurthii*	16	9	9	12.9
Bili	DRC	*P.t. schweinfurthii*	54	20	15	11.0
Gangu	DRC	*P.t. schweinfurthii*	6	0	0	0.0
Ituri	DRC	*P.t. schweinfurthii*	12	0	0	0.0
Kabogo	DRC	*P.t. schweinfurthii*	5	2	2	13.0
Maiko	DRC	*P.t. schweinfurthii*	1	0	0	0.0
Ngiri	DRC	*P.t. schweinfurthii*	8	0	0	0.0
Regomuki	DRC	*P.t. schweinfurthii*	2	1	0	9.0
Rubi-Tele	DRC	*P.t. schweinfurthii*	53	6	3	9.5
Tayna	DRC	*P.t. schweinfurthii*	3	1	1	12.0
Gishwati	Rwanda	*P.t. schweinfurthii*	132	24	24	13.3
Nyungwe	Rwanda	*P.t. schweinfurthii*	195	33	29	12.1
Issa	Tanzania	*P.t. schweinfurthii*	207	41	26	10.4
Budongo	Uganda	*P.t. schweinfurthii*	192	27	22	11.3
Bwindi	Uganda	*P.t. schweinfurthii*	155	36	29	11.3
Ngogo	Uganda	*P.t. schweinfurthii*	55	37	36	11.6
		Mean		17.35		9.66
		Mean excluding zeros		19.94		11.10

[1]Tai-ecotourism and Tai-R were combined and analysed as a single sampling location because of their close proximity (< 15 km).
[2]Bakoun and Sobory were combined and analysed as a single sampling location because of their close proximity (< 15 km).
[3]Comoe, Comoe-East and GEPRENAF were combined and analysed as a single sampling location because of their close proximity (< 15 km).
[4]Tweh, C. *et al*. Conservation status of chimpanzees *Pan troglodytes verus* and other large mammals in Liberia: A nationwide survey. *Oryx* **49,** 1-9 (2014)
[5]Murai, M. *et al*. Priority areas for large mammal conservation in Equatorial Guinea. *PloS one* **8,** 1-13 (2013)

Supplementary Table 1.4. Summary of genetics results categorized by subspecies populations.

Subspecies	Total Samples	Number of individuals typed at 7 or more loci	Number of individuals typed at 10 or more loci	Mean number of loci typed
P.t. verus	2808	523	428	11.36
P.t. ellioti	527	46	29	9.83
P.t. troglodytes	968	133	106	11.50
P.t. schweinfurthii	1094	237	196	11.50
Total	5397	939	759	11.34

Null alleles

Excess homozygosity in a locus suggests the possible presence of unaccounted-for null alleles and also leads to deviations from Hardy Weinberg equilibrium (HWE). However, under conditions of limited dispersal, a large population with widespread geographic distribution will typically display patterns of excess homozygosity due to geographic variability of allele frequencies among subpopulations. Additionally, unbalanced sampling within a population will lead to over and underrepresentation of subpopulations as well as gaps in the data. This combination of factors can lead to the possible detection of heterozygote deficiency. Tests of deviation from HWE and null alleles in a dataset under these conditions will lead to significant results due to the underlying stratification of allele frequencies, a phenomenon referred to as the Wahlund effect[13]. We performed tests of null allele frequencies and then subsequently verified whether or not the Wahlund effect was a valid explanation for any significant results. At the full-species scale, we only detected one locus for which the null allele frequency estimate was > 0.10 (D18s536) with a significant deviation from HWE ($p > 0.001$). When we performed separate tests of each subspecies population for this locus, all were in HWE and null allele frequencies were < 0.10, a clear sign of differing allele frequency

patterns among the subpopulations, i.e., the Wahlund effect. However, in analyses of *P.t. ellioti*, two other loci (D5s1457 and D3s3033) were observed to have null allele frequencies > 0.10. This issue likely arose from the small and biased sampling of the *P.t. ellioti* population, whereby one site (Gashaka) was considerably overrepresented, and was a consistent outlier in all population structure analyses. Additionally, there was also a locus (D5s1457) in the *P.t. schweinfurthii* population that displayed higher than expected homozygosity, for which the null allele frequency was > 0.10 with a significant deviation from HWE ($p > 0.001$). We suspected that a combination of another consistent outlier in our downstream analyses (Issa) and population-size effects (differences in N_e, i.e., the Wahlund effect) were driving this significant result. When excluding Issa, the null allele frequency was < 0.10, but deviations from HWE remained significant. When balancing the dataset (still excluding Issa) by randomly selecting a maximum of 15 individuals from each sampling location, the test for deviations from HWE was not significant. These results are in agreement with our observations of stratification in the *P.t. schweinfurthii* population in preliminary tests of population structure prior to sample size correction. We found that when the data were unbalanced, allele frequencies clustered among sampling locations, a phenomenon in this type of analysis similar to the Wahlund effect.

We conclude that variation in allele frequency patterns and N_e among and within subpopulations in our dataset led to the presence of the Wahlund effect, and are responsible for the positive detection of null alleles and deviations from HWE in two loci, one at the species level and one at the subspecies level. Importantly, the presence of unaccounted-for null alleles in the data decreases within-population diversity (lower

F'_{ST}) and increases between-site diversity (higher F'_{ST}) leading to observations of increased differentiation between populations, thus our downstream tests may be more likely to falsely detect spatial discontinuities in the data[13,14]. If our estimates of F'_{ST} are indeed affected by the presence of null alleles, it may, in part, explain the sharp divergence in the three-outlier populations in comparisons of nearby sampling locations. Consequently, incidental inflation of between-site diversity due to the possible presence of real null alleles does not invalidate our findings of connectivity across the species; rather it strengthens our results and supports our conclusions.

Relatedness

All of our analyses were potentially sensitive to oversampling of closely related individuals. Therefore, we sought to quantify the proportion of first-order relatives genotyped from each sampling location. Here we define first-order relatives as all individuals sharing at least one allele at each locus and with a parentage likelihood probability >0.95[15]. To accomplish this, we first performed a parentage simulation in Cervus[12], that assigns parentage probability based on allele frequencies, number of assumed candidate parents, proportion of assumed candidate parents in the sample, the proportion of loci typed and the proportion of loci mistyped. We then performed a maternity analysis in Cervus with sex set to “unknown”, such that all individuals could be potential “mothers” and all individuals could be “offspring”, and allowed for a single mismatch between parent and offspring. This approach assigned first-order dyads, regardless of sex, with 95% confidence. After removing all redundant reciprocal pairings, the total percentage of first-order related dyads sampled across all sites was

1.85%, which falls within the expected range[15,16], thus indicating a balanced, real-world representation of relatives in the sampled populations. However, when we examined the proportions by sampling location we identified several outliers (Supplementary Table 1.5). In particular, the proportion of first order assignments at Mt. Sangbé was over 3 standard deviations above the mean in our dataset. This high degree of relatedness, at least partially, explains why this population appears as an outlier in all of our analyses. Chimpanzees at Mt. Sangbé are isolated on a mountaintop with only 41 km^2 of forest cover as of 1995[17]. In addition, this population exhibits the lowest allelic diversity in our dataset, suggesting that they may have experienced a high level of inbreeding.

Supplementary Table 1.5. Proportion of first-order relatives identified and z score calculations for each sampling location with more than six genotypes.

Blue horizontal bars display relative differences in percentage of first-order assignments among sampling locations. Z scores highlighted in pink are sampling locations with first-order assignments higher than one standard deviation from the mean. The mean percentage (3.5%) of all sampling locations was used for standard deviation calculations for z scores. The overall mean was calculated from the total number of first-order assignments divided by the total number of dyads in the population.

Sampling locations	Country	*N* samples	*N* individuals	$\bar{x}$ loci typed	*N* First-order assignments	Total dyads	Percent first-order assignments	z score
Mt. Sangbé	Côte d'Ivoire	98	15	11.47	14	105	13.33%	3.21
Taï	Côte d'Ivoire	259	29	10.66	6	406	1.48%	-0.66
Djouroutou	Côte d'Ivoire	166	27	11.56	4	351	1.14%	-0.77
Comoé	Côte d'Ivoire	481	72	12.30	42	2556	1.64%	-0.60
Sangaredi	Guinea	116	17	10.76	0	136	0.00%	-1.14
Bakoun-Sobory	Guinea	274	69	11.57	20	2346	0.85%	-0.86
Sobeya	Guinea	228	46	10.15	11	1035	1.06%	-0.79
Boe	Guinea Bissau	118	72	11.03	9	2556	0.35%	-1.03
East Nimba	Liberia	137	14	11.71	3	91	3.30%	-0.07
Sapo	Liberia	224	17	10.00	5	136	3.68%	0.06
Grebo	Liberia	200	23	10.91	8	253	3.16%	-0.11
Bafing	Mali	107	15	12.67	9	105	8.57%	1.66
Dindefelo	Senegal	184	58	12.62	33	1653	2.00%	-0.49
Kayan	Senegal	76	15	11.40	7	105	6.67%	1.03
Outamba-Kilimi	Sierra Leone	90	24	10.00	4	276	1.45%	-0.67
Korup	Cameroon	125	7	8.71	2	21	9.52%	1.97
Gashaka	Nigeria	102	30	10.03	20	435	4.60%	0.36
Conkouati	Congo	140	17	10.12	5	136	3.68%	0.06
Goualougo	Congo	92	49	12.16	19	1176	1.62%	-0.61
Mts de Cristal	Gabon	136	9	13.00	2	36	5.56%	0.67
Loango	Gabon	145	12	11.83	2	66	3.03%	-0.15
Lope	Gabon	139	29	11.41	10	406	2.46%	-0.34
Bili	DRC	62	20	11.00	2	190	1.05%	-0.80
Gishwati	Rwanda	132	24	13.25	21	276	7.61%	1.34
Nyungwe	Rwanda	195	33	12.09	18	528	3.41%	-0.03
Issa	Tanzania	207	41	10.44	32	820	3.90%	0.13
Budongo	Uganda	192	27	11.26	9	351	2.56%	-0.30
Ngogo	Uganda	55	37	11.65	12	666	1.80%	-0.55
Bwindi	Uganda	167	36	11.28	12	630	1.90%	-0.52
Overall		4290	840	304.93	321	17336	1.85%	-0.09

Supplementary Note 2

Summary: We used both spatial and non-spatial approaches to identify geographic stratification of genetic data in chimpanzees. Using spatially agnostic analyses we found population structure in accordance with the currently held subspecies divisions; however, this pattern also correlated closely with the spatial sampling gaps in our data, similar to previous studies. Linear regressions of all categories of within- and between-group comparisons revealed that between-subspecies comparisons were within the range of within-subspecies patterns, with the exception of those involving *P.t. verus*, whereby presumptive allele saturation was influencing the behaviour of the functions. Finally, we tested whether or not the effective barriers detected in our species-scale spatially explicit analysis (EEMS) were a result of highly localized differentiation. We re-analysed our data excluding three sites associated with the barriers. We found that the barriers were no longer significant, suggesting that they were a result of local differentiation and no major discontinuities were present in the genetic data.

Cluster (STRUCTURE) analyses

We used STRUCTURE version 2.3.4[18], a “spatially agnostic” Bayesian cluster analysis, to test for the presence of geographic stratification in our genetic data. This program utilizes allele frequency and linkage equilibrium patterns to assign individual genotypes to a user-defined number of groups known as clusters (*K*). Then a test is performed to ascertain which *K* best explain the data. This algorithm is sensitive to the presence of isolation by distance (IBD; spatial auto correlation in the genetic data), a bias the

authors noted in the user manual[18]. Spatially agnostic analyses rely on uniform geographic distribution of the genetic data, which is critical in cases in which IBD is present in the data, a violation of which will lead to a considerable risk of a Type I error, a well-known limitation[18-21]. Another important consideration is unbalanced sampling, whereby relative over and underrepresentation of the genetic diversity among subpopulations (variation in sampling density) will considerably affect allele frequency calculations, leading to the false detection of population structure[22]. Finally, comparing populations consisting of different effective population sizes (N_e) increases the risk of a Type I error simply because it replicates the problem of variation in sampling density[22]. Indeed, our dataset violates all of these model assumptions: (1) *P.t. verus* is overrepresented, while *P.t. ellioti* is underrepresented (Supplementary Table 1.3, 1.4), (2), IBD has been shown to be present in chimpanzees[19,20], (3) it is well established that *P.t. verus* has a considerably smaller N_e than the other three subspecies populations[23,24] and (4) despite our best efforts, our genetic sampling is spatially heterogeneous. Although it is possible to create a subset of *P.t. verus* from our dataset to minimize the unbalanced sampling (which we performed), we cannot account for overrepresentation due to their low N_e. For example, if some equal number of samples were randomly drawn from each subspecies population, creating a perfectly balanced dataset, *P.t. verus* will always have more of their total diversity sampled than the other populations, and, hence, be overrepresented in the data. It is conceivable that different N_e can be accounted for by adjusting the number of draws from each sample population proportional to their respective N_e; however, we contend that it should not be done in

this case as it is a relevant feature of the population, despite the challenges of the inherent biases.

Since spatially agnostic analyses, such as STRUCTURE, have been used in all previous studies of chimpanzee population structure, we were interested in comparing our results to previous results despite the outlined limitations and its inappropriate application to our dataset. This analysis is useful as it is potentially helpful in visualizing some of the features of the populations being compared, *e.g.*, admixture and overall distribution of diversity, but it is important to consider the limitations in inferences of K when assumptions are violated. Nevertheless, because of the sampling assumption violations in our data the results of these analyses are spurious and therefore unreliable.

For our analyses, we assumed the allele frequencies were correlated and admixture had occurred among populations. To ensure the model reached stability we performed 100,000 burn-in steps followed by 1,000,000 data collection steps. For all analyses we imposed from 1 to 8 clusters (K) within the sample population at 20 iterations for each value of K. We used both $Pr(X \mid K)$[25] and ΔK[26] methods for inferring the number of K clusters.

Following genotype reconstruction and prior to performing any other analyses it was necessary to identify contaminated or misidentified samples. Although many primate species occur sympatrically within the chimpanzee range, they are not closely related and therefore not expected to share many alleles. Alleles from monkeys will tend to have unusual sizes, falling outside chimpanzee allele bins and ranges. Closely related species, such as humans and gorillas, which also occur sympatrically, however are

much more likely to share alleles, making it more difficult to detect them among the genotypes in our dataset. Also, it is considerably more difficult to distinguish faeces among these three species as compared to between apes and monkeys. Since the STRUCTURE clustering algorithm enables us to detect population structure within a species, it is also effective for detecting interspecific structure as well[27]. To test for the presence of contamination from closely-related sympatric species we added 10 known human and 10 known gorilla samples to our dataset for analysis of $K = 3$. We were able to identify seven gorilla samples in areas where they are known to co-occur with chimpanzees. We also detected four other gorilla samples in West Africa where they are absent, suggesting that they likely originated from monkey species. Fifty genotypes clustered with the 10 known human genotypes, nearly all of which originated from three field sites located in Côte d'Ivoire (Djouroutou, Taï, and Comoé). This suggests localized sampling issues rather than a widespread problem in our sampling approach. Importantly, we did not include monkey reference samples in this analysis, such that the species attributions of the samples clustering with the gorilla and human genotypes are not certain, other than they are not of chimpanzee origin. All these suspected non-chimpanzee samples were removed and excluded from all downstream analyses.

In order to proceed with the cluster analysis, it was necessary to account for imbalances and spatial heterogeneity in our data by creating random and balanced subsets of the genotypes. We limited our analyses to individuals with a minimum of seven confirmed loci, and to prevent overrepresentation of genetically similar individuals, we limited the maximum number of individuals from any one site to 20. We employed a two-level strategy of subsetting our data, in which we first randomly sampled 20 genotypes from

sites with more than 20 individuals. We performed 10 iterations of subsetting at this level. Then we subsequently pooled all of the selected genotypes by subspecies, followed by randomly sampling 70 candidates from each population, and repeated this step for a total of ten randomized iterations. Our data include 46 genotypes from *P.t. ellioti*, with 30 of those originating from a single site (Gashaka). To mitigate this, we further down-sampled Gashaka by randomly selecting 10 genotypes from this location; this however increased uneven sampling of *P.t. ellioti* relative to the other subspecies populations. For each iteration of this analysis, this approach yielded a total of 10 different randomly sampled populations from our dataset, comprising 70 different individuals each from *P.t. verus*, *P.t. troglodytes*, *P.t. schweinfurthii* and 36 individuals from *P.t. ellioti.*

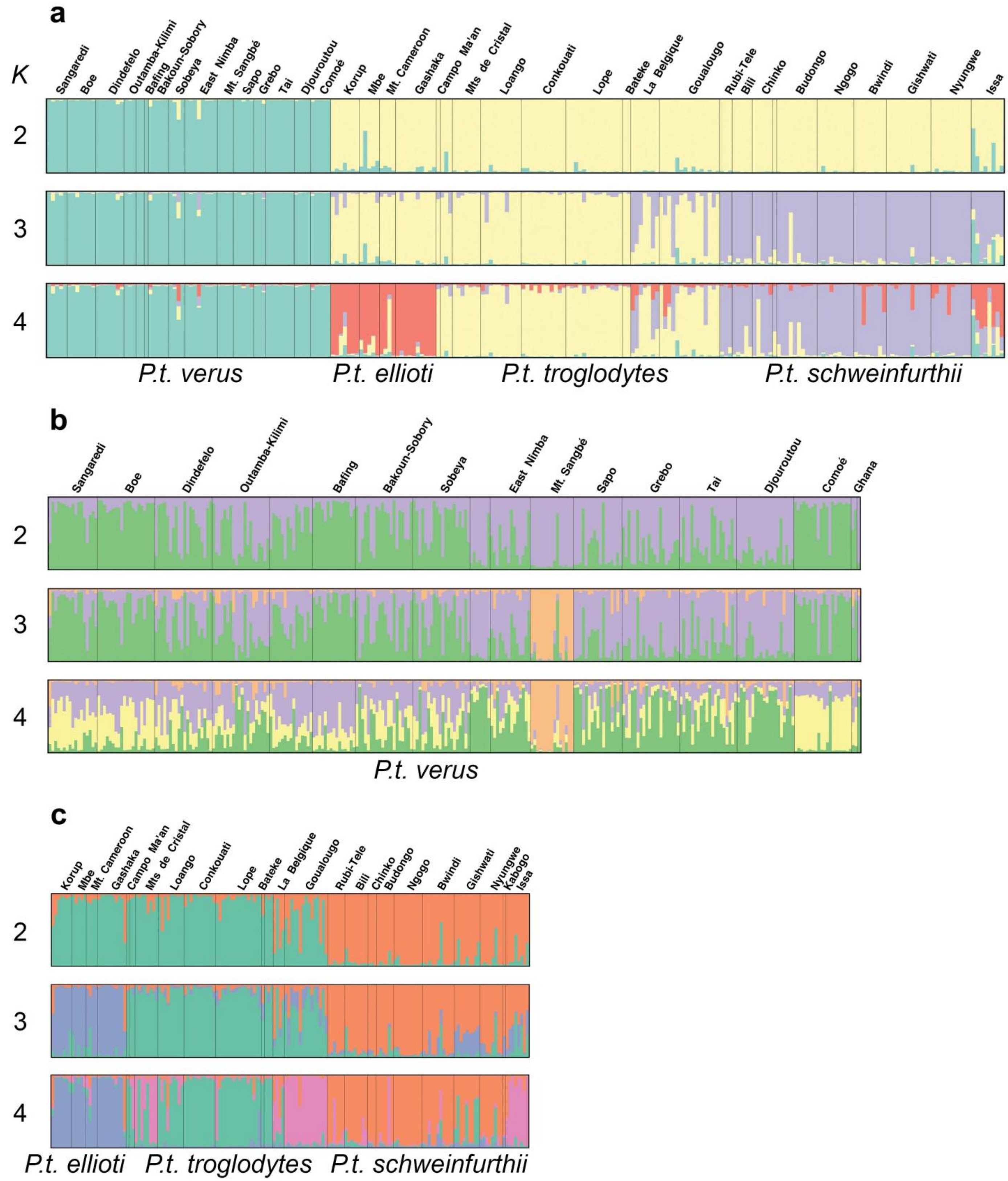
a
K
Sangaredi
Boe
Dindefelo
Outamba-Kilimi
Bafing
Bakoun-Sobory
Sobeya
East Nimba
Mt. Sangbé
Sapo
Grebo
Tai
Djouroutou
Comoé
Korup
Mbe
Mt. Cameroon
Gashaka
Campo Ma'an
Mts de Cristal
Loango
Conkouati
Lope
Bateke
La Belgique
Goualougo
Rubi-Tele
Bili
Chinko
Budongo
Ngogo
Bwindi
Gishwati
Nyungwe
Issa
2
3
4
P.t. verus
P.t. ellioti
P.t. troglodytes
P.t. schweinfurthii
b
Sangaredi
Boe
Dindefelo
Outamba-Kilimi
Bafing
Bakoun-Sobory
Sobeya
East Nimba
Mt. Sangbé
Sapo
Grebo
Tai
Djouroutou
Comoé
Ghana
2
3
4
P.t. verus
c
Korup
Mbe
Mt. Cameroon
Gashaka
Campo Ma'an
Mts de Cristal
Loango
Conkouati
Lope
Bateke
La Belgique
Goualougo
Rubi-Tele
Bili
Chinko
Budongo
Ngogo
Bwindi
Gishwati
Nyungwe
Kabogo
Issa
2
3
4
P.t. ellioti
P.t. troglodytes
P.t. schweinfurthii

389

Supplementary Figure 2. STRUCTURE group assignment plots. (**a**) Example of group assignment plot (*K* = 2 – 4) for all sampling locations arranged from west to east with the two-level subsetting method applied (*n* = 236). This is one of 10 iterations of subsetting. Although the support for *K* varied, the patterns in the plot were consistent at all hierarchical levels. *K* = 2, with *Pan troglodytes verus* as 1 and *P.t. ellioti*, *P.t. troglodytes* and *P.t. schweinfurthii* (collectively *ETS*) as the 2nd group was optimal in 7 of 10 subset iterations (range = 2 – 7). (**b**) Group assignment plots for *P.t. verus* sampling locations (*K* = 2 – 4) using site-level subsetting (*n* = 283). From two to five clusters were supported depending on which iteration of the subsetted data was analysed. Increasing *K* only further subdivides group membership within individuals, as is already evident in *K* = 4. (**c**) Group assignment plots for *ETS* (*K* = 2 – 4) genotypes from the subsetted full dataset (*n* = 176). From two to five clusters were supported depending on which iteration of the subsetted data was analysed. Clustering along the subspecies divisions are influenced by the overrepresentation of *P.t. verus* samples in our data, the underrepresentation of *P.t. ellioti* samples, and large sampling gaps between *P.t. verus* and *P.t. ellioti* (1656 km) and between *P.t. troglodytes* and *P.t. schweinfurthii* (987 km) in the presence of isolation by distance (IBD). Discontinuous sampling in the presence of IBD is known to result in an overestimation of genetic structure. Despite the clustering along subspecies divisions, we also observe *P. t. troglodytes* individuals belonging to the "*ellioti*" and "*schweinfurthii*" clusters, and vice versa. Furthermore, the clustering in these plots aligns with the sampling gaps depicted in Supplementary Figure 3c.

We found that every iteration yielded similar results across all analyses with some subtle fluctuations in group assignments, but inferences of *K* varied considerably. At the species scale, there was strong support for *K* = 2 in most iterations (7/10; range = 2–7; see Supplementary Table 2.1 for full account) when using Δ*K*, and group assignments consistently segregated into the same two discrete clusters (Extended Data Fig. 5a) with minimal admixture, in which *P.t. verus* formed one cluster and the combination of *P.t. ellioti*, *P.t. troglodytes* and *P.t schweinfurthii* (henceforth collectively referred to as *ETS*) formed the second cluster. The differentiation observed here is likely influenced by the large geographic gap between sampled populations (ca. 1650 km) and low genetic diversity in *P.t. verus*, in which nucleotide diversity has been shown to be over two-fold lower than in *P.t. troglodytes*[23]. Notably, at *K* = 2, there was no detectable evidence of a comparable division among the other three subspecies populations in the *ETS* cluster. However, when viewing *K* = 3 and *K* = 4 at the species scale, we consistently see evidence of substructure, suggesting that the signal of differentiation between *P.t. verus* and *ETS* overwhelms any possible signal of *ETS* differentiation in our dataset. The optimal value of LnP(*K*) was either found to be *K* = 7 or *K* = 8 clusters, which always forms a split between *P.t. verus* and some combination of another six or seven clusters within *ETS*. LnP(*K*) plots all approached their asymptote at *K* = 8. Since the Evanno method is limited to the highest hierarchical level of structure, it was necessary to analyse both the *P.t. verus* and *ETS* populations independently.

The *P.t. verus* population yielded an interesting pattern of substructure where one population appeared to be somewhat unusual. Mt. Sangbé consistently behaved as a discrete cluster from the rest of the population at any value above *K* = 2. This result was

not unexpected given the high proportion of first-order relatives we identified in our parentage analysis (over three standard deviations above the mean; Supplementary Table 1.5). Outside of Mt. Sangbé, and in some iterations Comoé (usually from K = 5 and higher), individuals across the *P.t. verus* populations displayed fractional group assignments, with no other sites appearing to be differentiated at any K. The optimal value of K varied between two and seven clusters across sampling subsets, but, apart from Comoé and Mt. Sangbé, group assignment among the other sites did not segregate into geographically meaningful clusters.

Supplementary Table 2.1. L_nP(*K*) and Δ*K* results for all STRUCTURE analyses for each subset iteration. Inferred number of clusters (*K*) for each iteration for the entire species range (*Pan*), *Pan troglodytes verus* only, and the combination of *P.t. ellioti*, *P.t. troglodytes*, and *P.t. schweinfurthi* (collectively *ETS*).

Iteration	Pan LnP(*K*)	Pan Δ*K*	*P.t. verus* LnP(*K*)	*P.t. verus* Δ*K*	*ETS* LnP(*K*)	*ETS* Δ*K*
1	8	2	5	5	8	3
2	7	2	5	3	6	2
3	7	7	5	4	6	6
4	7	2	5	5	7	3
5	7	4	7	7	6	3
6	8	2	5	4	8	6
7	8	2	5	5	8	2
8	8	2	7	7	6	3
9	8	2	5	2	8	3
10	8	4	7	7	7	3
Range	7 - 8	2 - 7	5 - 7	2 - 7	6 - 8	2 - 6
Mean	7.60	2.90	5.60	4.90	7.00	3.40
Standard Deviation	± 0.49	± 1.58	± 0.92	± 1.64	± 0.89	± 1.36
Median	8.00	2.00	5.00	5.00	7.00	3.00

Within *ETS*, *P.t. ellioti*, *P.t. troglodytes* and *P.t. schweinfurthii* are considered historically and geographically discrete populations and have subspecies designations; however, the species-wide cluster analysis assigned them to a single cluster. When analysed independently of *P.t. verus*, the optimal number of clusters for *ETS* varied between two and six in the different subsetted datasets, with *K* = 3 being optimal most often (6/10; Supplementary Table 2.1). However, the group-assignment plot for *K* = 3 does not display discrete clustering. In all iterations, we see the same pattern in which each subspecies is generally sorted into its own cluster, but with multiple individuals exhibiting admixture from the other subspecies in a pattern that occurs along a mostly longitudinal gradient. This is particularly noteworthy for two reasons. (1) We have an extensive sampling gap (ca. 987 km) between the easternmost population of *P.t. troglodytes* (Goualougo) and the westernmost population of *P.t. schweinfurthii* (Bili)

(refer to Supplementary Figure 3a, c). Nevertheless, we find a signal of admixture of *P.t. schweinfurthii* in the population of Goualougo, and even more so in individuals from La Belgique, in which they bear nearly equal admixture from both *P.t. troglodytes* and *P.t. schweinfurthii*, despite being located further west than Goualougo. This suggests possible admixture from the north where populations in the Central African Republic have only recently been extirpated and were likely continuous to the Republic of Congo. Moreover, this result suggests historical gene flow may have been occurring around the Ubangi River as opposed to directly across it. (2) *P.t. ellioti* is drastically underrepresented in the data. Not only did we have too few samples from the *P.t. ellioti* population, but we subsetted the Gashaka samples to prevent those genotypes from being over-represented in the data. These severely unbalanced data notwithstanding, we still observed admixture between *P.t. ellioti and P.t. troglodytes*, which has been previously shown to some degree with a more robust *P.t. ellioti* dataset[28]. We also performed an analysis of *P.t. troglodytes* and *P.t. schweinfurthii* (data not shown) and found that the group assignment plots were qualitatively similar to Supplementary Figure 2a and c, in which an admixture gradient between the two subspecies is evident. These results demonstrate the characteristic sensitivity of the STRUCTURE software to sampling biases and stochasticity even despite our best efforts to mitigate them.

Notably, these results clearly correspond with the patterns we observed in the heat matrices (Supplementary Figure 3a, b), in which geographic sampling gaps in the presence of IBD cannot be excluded from explanations of the spatial stratification detected by these analyses in the genetic data.

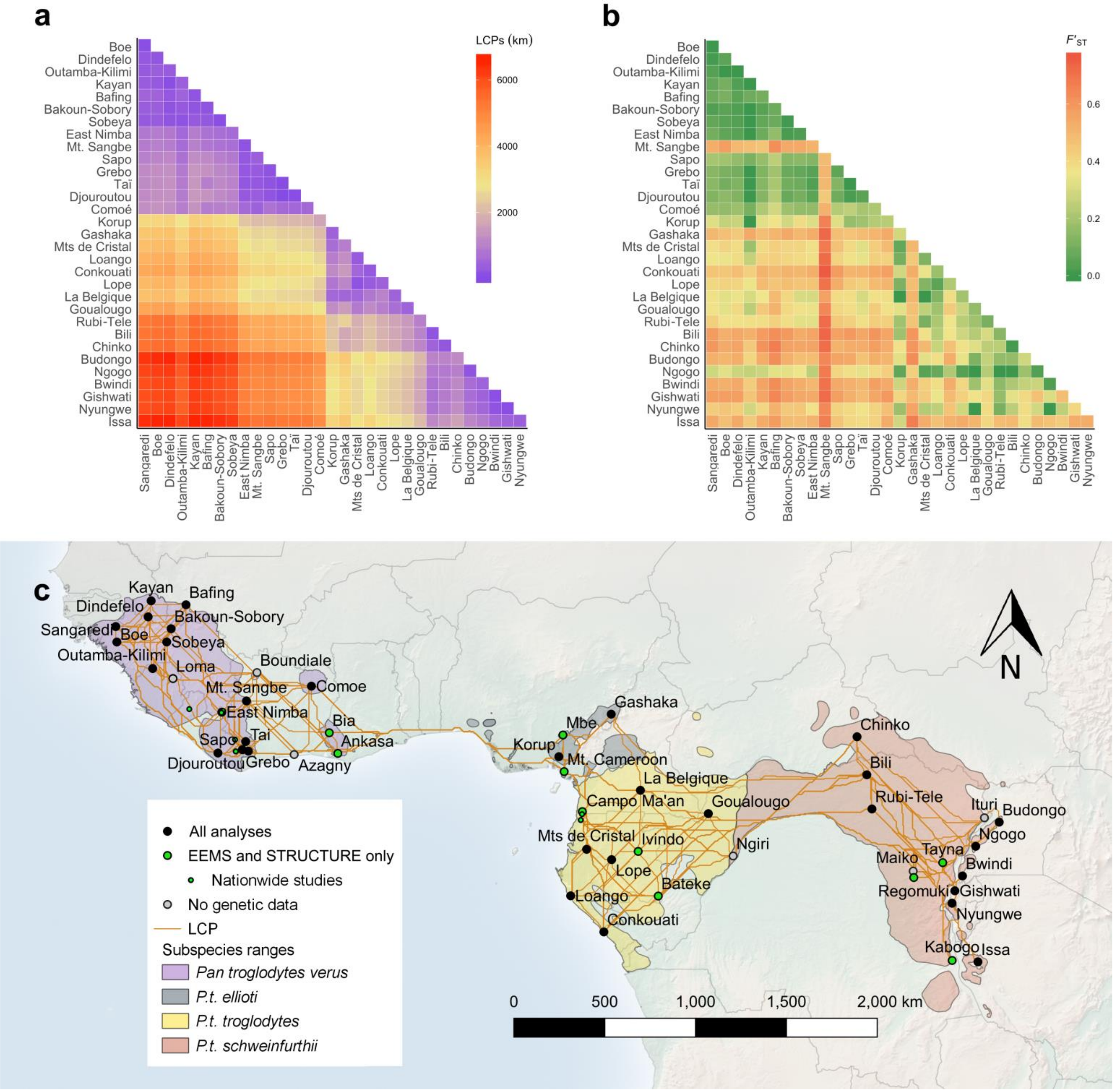

Supplementary Figure 3. Least cost paths (LCPs) and genetic diversity (F'_{ST}) heat matrices and LCP map of PanAf sampling locations. (**a**) Heat matrix of pairwise LCPs between all sites with a minimum of six individuals typed at seven or more loci.

Sharp transitions in colour coincide with areas where large sampling gaps occur. (**b**) Heat matrix of pairwise F'_{ST} values of all sampling locations with a minimum of six individuals typed at seven or more loci, in which stark colour contrast highlight areas of strong genetic differentiation. These heat matrices demonstrate how the overall patterns observed in our genetic data correspond closely to the geographic data, apart from three notable outliers sampling locations (Mt. Sangbé, Gashaka and Issa). (**c**) Map of all locations where samples were collected for the present study as part of the PanAf. LCPs are presented here to display the pairwise distances that were used for analyses involving geographic distances. Locations with solid black circles were used in all analyses, locations with green-filled circles were used for EEMS and STRUCTURE analyses only, and locations depicted by grey-filled circles did not have any usable gentoypes. Small green circles represent samples collected as part of nationwide studies in Liberia and Equatorial Guinea.

Isolation by distance (IBD) and distance estimators

To determine whether the current delineation of subspecies is supported by genetic evidence, we first tested for collinearity between genetic and geographic distance, IBD. For an estimator of genetic distance, we used F'_{ST}, a measure standardizing genetic differentiation by its maximum possible value given the population diversities. Standard F_{ST} is sensitive to differences in within-population diversity[29]. F'_{ST} was ideal for this study because our dataset compares populations consisting of different effective sizes (N_e), and the microsatellites we used are composed of highly polymorphic loci with different mutation models and rates (see Supplementary Figure 3b for pairwise F'_{ST} matrix). To generate a balanced dataset to minimize overrepresentation of densely sampled locations we imposed an upper limit of 20 randomly drawn samples per site, and a lower limit of 6. We then used Arlequin software version 3.5.2.2[30] on the resulting dataset to calculate F'_{ST}, by dividing F'_{ST} by maximum F'_{ST} (F'_{STMax}). F'_{STMax} was obtained by assigning a unique set of allele names to each allele for all loci for each sampling location and then recalculating F_{ST}. For geographic distances we generated

least cost paths (LCP) to approximate actual distances between sampling locations (Supplementary Figure 3a, c), but more importantly to force distance measurements to account for the presence of large water bodies, such as the Atlantic Ocean. We plotted a heat matrix pairwise LCPs and found that the pattern closely matched that of the F'_{ST} heat matrix (Supplementary Figure 3a-b), suggesting that our geographic sampling gaps highly correspond with differentiation present in genetic distances we observed in the data. Three divergent outliers of interest, Mt. Sangbé, Gashaka and Issa, stood in stark contrast to the overall pattern of diversity in Supplementary Figure 3b.

Stratified and partial Mantel tests

Mantel tests are useful to assess the presence of spatial autocorrelation and collinearity between two data sets. This analysis in its simplest form compares two matrices, x and y, of identical dimensions, in which a permutation test is performed, meanwhile maintaining the dyadic structure of the data[31]. Modifications allow for tests of both IBD and hierarchical clustering. Though IBD has previously been shown to be present in chimpanzee genetic data and it is ubiquitous in sexually reproducing and dispersing populations, it was still necessary to test for its presence in our dataset to validate our inferences and satisfy downstream model assumptions. To perform all Mantel tests we used the mantel function in the R package, “vegan” version 2[32]. To assess IBD in the genetic data we needed to account for the presence of hierarchical structure in the data. To do so we applied a stratified Mantel test, whereby the matrix of genetic distance is permuted within predefined clusters, in our case subspecies population[33]. Note, the result of this test is sensitive to correct group assignment of the data. The presence of

IBD was rejected using the four-subspecies model (Mantel's $r = 0.638$, $p = 0.323$). Since the STRUCTURE results at the species level consistently supported differentiation between *P.t. verus* and *ETS*, we ran a second stratified Mantel test using these two populations. We found that this yielded a trend in agreement with the cluster analysis results (Mantel's $r = 0.645$, $p = 0.081$). This test therefore appeared to be influenced by how we defined the groups so we used a partial Mantel test to assess collinearity between genetic and geographic distance, while controlling for binary cluster membership in predefined groups, in our case subspecies. Note, in tests of IBD applied in this fashion, this approach has been shown to have a 20% Type I error rate (though, typically showing an almost zero effect size), but a 0% Type II error rate[33]. The result of this test supported the presence of IBD with a large effect size (Mantel's $r = 0.520$, $p = 0.001$). The large effect size suggests this was likely not a false positive result. Overall, these tests converged on supporting the presence of IBD. Since IBD is the widespread and standard pattern of genetic diversity in populations, and has been previously shown to be present in chimpanzee genetic data, we conclude the four-subspecies model of population structure is problematic in our dataset in these tests of IBD, suggesting that a number other than four discrete populations characterize these data.

Next we tested for hierarchical clustering of the genetic data as defined by the chimpanzee subspecies delineation and presumed barriers to dispersal ($K = 4$). Since spatial autocorrelation of our genetic data was significant, it was necessary to account for it in the model. Here we again used a partial Mantel test comparing genetic data (x) to *a priori* defined binary cluster membership (y) and geographic distance (z), to control for IBD. Our test of hierarchical population structure was significant with a moderate

effect size (Mantel's $r = 0.251$, $p = 0.011$), indicating that stratification ($K = 4$) was present in our data. Importantly, however, there is a relevant limitation of interest for our study; this test can only identify the overall effect size among the populations being compared and not between-population effect size. So, one highly differentiated population could drive a significant result, while the other populations may not actually be meaningfully differentiated from each other. The STRUCTURE results indicated strong differentiation between *P.t. verus* and *ETS*, therefore to see if this drove the significant result in the partial Mantel test of $K = 4$ we performed a second test on *ETS* only ($K = 3$). Here we found no support for differentiation among the three subspecies comprising *ETS* (Mantel's $r = -0.065$, $p = 0.633$). Finally, we performed a partial Mantel test comparing *P.t. verus* to *ETS* to quantify the effect size between these two populations (Mantel's $r = 0.253$, $p = 0.001$). Interestingly, the effect size was the same as the four-subspecies model, but the *p*-value was ten-fold smaller. We concluded that statistical support for the four-subspecies model in the partial Mantel test was driven by strong differentiation in the species between *P.t. verus* and *ETS,* and as there was no change in effect size, the differentiation among *ETS* did not meaningfully contribute to the significant result in $K = 4$. Importantly, both the IBD and within-*ETS* tests support the presence of genetic connectivity among the *ETS* populations.

Linear regressions

To quantify the extent to which geographic distance (*D*) explains genetic distance (*G*) and to ascertain the effect of the divergent outliers detected in the F'_{ST} matrix, we fitted regressions to all sites with at least six individuals typed at seven or more loci (Main

text; Fig 2.a). For these tests we transformed genetic distance to $F'_{ST}/(1-F'_{ST})$ since this transformation is expected to have a linear relationship with distance[34]. Strict IBD appears as a gradient in which *G* increases linearly with *D*. Detectable departures from this pattern, e.g., clusters that do not fall along the cline, are genetically differentiated and signify the historical presence of physical or behavioural barriers that reduced reproductive potential between populations. Geographically, it is widely held that several major physical barriers to chimpanzee dispersal are present within their range, which are presumed to have given rise to the four currently described subspecies. Although there is not a unified consensus on the definition or concept of subspecies, the assessment of population structure and detection of genetically distinct and/or isolated populations is very informative. Failing to find evidence of genetic discontinuity between or among subspecies populations would suggest recent gene flow has occurred and may currently persist.

As detailed in the main text, we fitted regressions of $G \sim D$ to the entire dataset, to the dataset with outliers excluded and then also highlighted within and between-subspecies comparisons (Fig. 2a – d). We found a significant linear correlation with moderate effect size when all sites were included. When three consistent outlier sampling locations in all analyses (Mt. Sangbé, Gashaka and Issa) were excluded from the model, the effect size nearly doubled to become strong, illustrating the considerable influence of local effects (in only 3 sites) on the species-wide pattern (Fig. 2a). We also found that, on average, the genetic distance between two pairs of locations is higher (given the same geographic distance) between subspecies than within subspecies (Fig. 2b). As the partial Mantel test of four discrete subspecies populations does not specifically reveal

which, if not all, comparisons within chimpanzees is driving the significance of the test, we fitted separate linear regression functions to all within- and between-subspecies comparisons at the subspecies-group level (Fig. 2c, d). The slope of these functions explains the strength of the effect of geographic distance on genetic distance, while the y-intercept values indicate within-sampling location diversity (the estimated level of diversity of the comparison pair if geographic distance between them were zero). Although this is not a formal test, here we found variability in the slope and intercept of the *G* ~ *D* linear regression in within- and between-subspecies combinations (Supplementary Figure 2b), thus confirming that the four-subspecies partial Mantel test is unable to account for this relevant feature of the data. This issue is particularly important given the overrepresentation of *P.t. verus* and the underrepresentation of *P.t. ellioti* samples. The same-subspecies pairs in the full dataset are largely *P.t. verus* – *P.t. verus* pairs and the 'different subspecies' pairs are largely *P.t. verus* – *P.t. troglodytes* or *P.t. verus* – *P.t. schweinfurthii*. The *P.t. verus* – *P.t. verus* regression is divergent from the *P.t. verus* – *P.t. troglodytes* and *P.t.verus* – *P.t. schweinfurthii* regressions (Fig. 2b – d), which likely drives the results of the partial Mantel test we performed. These figures also clearly show the expected asymptotic behaviour of the *G* ~ *D* regression as distances between *P.t. verus* and the other subspecies increases. Furthermore, the *P.t. troglodytes* – *P.t. schweinfurthii* regression is much more similar to the within-subspecies regressions than to the *P.t. verus* – *P.t. troglodytes* and *P.t. verus* – *P.t. schweinfurthii* regressions. The small sample sizes available for regressions involving *P.t. elliotti* (only two locations: Korup and Gashaka) are reflected by the large standard errors visible in Fig. 2d. However, the estimated intercepts and slopes for these

regressions are consistent with the general asymptotic pattern, except for the *P.t. elliotti* – *P.t. troglodytes* pairs. Note, however, that this regression is computed on Korup – *P.t. troglodytes* and Gashaka – *P.t. troglodytes* pairs only. Korup is geographically close and genetically similar to most *P.t. troglodytes* locations (Supplementary Figure 3a – c), while Gashaka is more geographically distant from *P.t. troglodytes* locations and is one of those few locations showing extreme genetic differentiation regardless of geographic proximity (Fig. 2a; Supplementary Figure 3b, 4a, b). Therefore, the uniqueness of the *P.t. elliotti* – *P.t. troglodytes* regression appears to be entirely due to the uniqueness of the Gashaka genotypes. In conclusion, these comparisons of $G \sim D$ did not provide evidence of any genetic discontinuities or barriers to gene flow across the chimpanzee range consistent with the currently recognized subspecies delineation, though differentiation, stemming at least in part from low N_e, was apparent in the *P.t.verus* population.

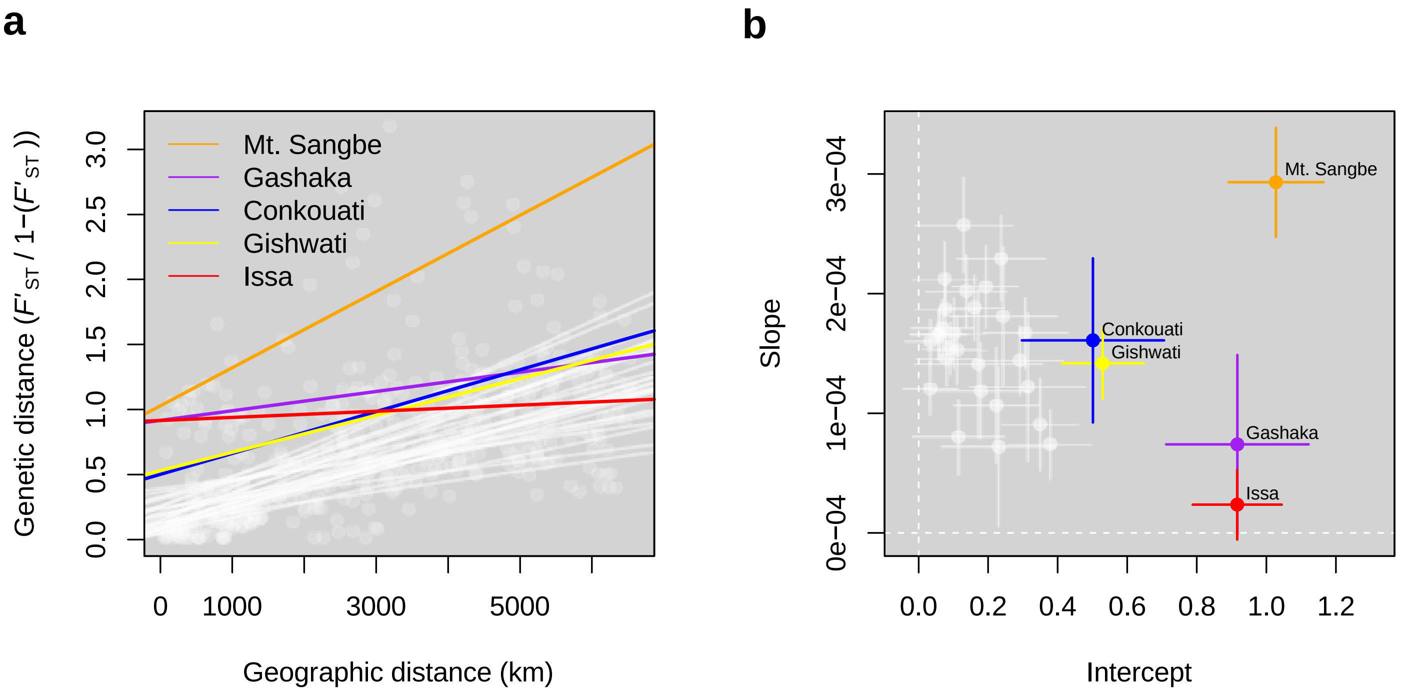

Supplementary Figure 4. Outlier detection in site level linear regressions. (**a**) Regression lines of pairwise comparisons of genetic distance as a function of geographic distance, whereby each line represents a single site compared to all other sites. Coloured lines highlight outlier sampling locations. (**b**) Intercept versus slope plot of site-level regression functions. The horizontal and vertical bars represent standard error of the mean. Coloured outlier sites display increased relative y-intercept values indicating higher-than-average levels of differentiation in comparison with nearby sites (i.e., strongly localized differentiation). Gashaka and Issa show similar relative genetic distance (low slope) regardless of geographic distance but a high intercept value, signifying consistently high differentiation in comparison to all other sites. Meanwhile, although Mt. Sangbé displays high differentiation in comparisons to all other sites, this effect increases with distance.

Detection of a population bottleneck in P.t. verus

We observed that allele size ranges in the genotypes from the *P.t. verus* population tended to nest within the allele-size range of the other subspecies populations, in particular, *P.t. troglodytes,* which is considered to be the oldest[24]. Such a pattern would emerge in cases where a founder population diverged from a larger population as genetic drift is expected to reduce allelic diversity in the former as new alleles have not

yet accumulated through mutation processes. Similarly, population bottlenecks will also reduce diversity due to drift and inbreeding. Since the split between *P.t. verus* and *P.t. troglodytes* is considered to be the deepest among the subspecies populations, occurring up to some 800 ka[24], and microsatellites have high mutation rates, which flatten ancient patterns of demography, the possibility of these observations being a signal from the original divergence between these two populations is extremely unlikely. Thus either the split between these two populations occurred more recently then has been previously shown or *P.t. verus* may have a complex history of expansions and contractions with extended periods of both isolation and sustained gene flow. Since our genetic data have multiple mutation models and population contraction/expansion analyses using microsatellite data assume strict stepwise mutation (SMM), we were unable to perform a formal test. Nevertheless, we explored other possible methods to further evaluate our dataset for signals of a bottleneck (and presumptive recent expansion) by assessing allele patterns using simulated data.

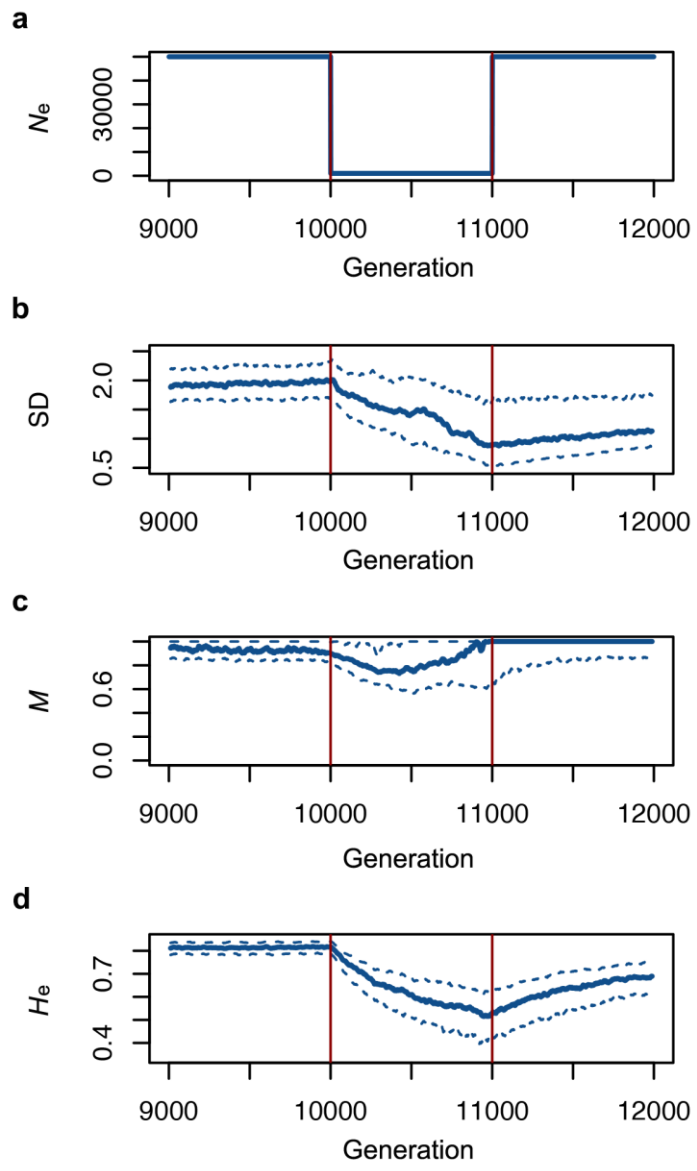

Supplementary Figure 5. Variation in standard deviation (SD) of mean allele sizes, Garza-Williamson index (*M*), and expected heterozygosity (H_e) in response to simulated population bottlenecks. (**a**) A simulated population of 50,000 individuals that experienced a contraction to 1,000 individuals lasting 1,000 generations followed by a subsequent full recovery. The response to simulated population bottleneck and full recovery in SD (**b**), *M* (**c**), and H_e (**d**). Plots b, c and d show medians (solid lines) with 0.25 and 0.75 quantiles (dashed lines) from 100 simulations, each replicating a single locus with the same mutation rate and mutation model. SD has a slow decline and increases slowly after full N_e (effective population size) recovery and does not reach pre-bottleneck levels after 1,000 generations. H_e also displays a slow gradual decline, but responds quickly to the recovery of N_e. Conversely, *M* begins to increase well before population recovery, and then exceeds initial pre-bottleneck levels. These

simulations suggest that we may infer populations displaying relatively low SD combined with a high *M* are likely to have undergone a bottleneck. Similar inferences from comparing H_e to *M* may be drawn, although H_e responds more quickly to recovery of N_e, and is, therefore, less likely to retain the signal as time increases.

First, we simulated a population demographic history with an initial N_e of 50,000 for 10,000 generations, followed by a bottleneck (N_e = 1,000), which lasted for 1,000 generations, then a subsequent full recovery which extended for 50,000 generations. We performed 100 simulations, each representing a single locus, using the same mutation rate and strict SMM for each run. We then calculated the mean standard deviation of allele size (SD), expected heterozygosity (H_e) and estimated the mean Garza-Williamson's *M* for each generation and plotted them (Supplementary Figure 5a – d). *M* is an index of allelic diversity, which is used to detect population contraction and is a ratio of the number of alleles (*k*; numerator) in proportion to the allele size range (*r*; denominator)[35]. Typically, after a bottleneck, many alleles are lost due to random drift and k becomes smaller, while initially, the range (*r*) is likely to remain large. As alleles at the extreme ends of the range tend to be rare, they also become lost due to random drift, leading to a decrease in *r*, leading to a positive slope of *M*. As new alleles accumulate through new mutations, thereby increasing *k*, the slope of *M* becomes steeper, especially if alleles at the extreme ends of the range continue to be lost due to random drift. Importantly, this method assumes a strict SMM, a violation of which will affect *M*. In our dataset, violations of strict SMM are mostly single BP indels, which increase the number of alleles in a given range, leading to inflated values of *M*. SD describes the spread and peak of allele-size ranges. A large effective population in equilibrium will have a high SD as the allele ranges will be wide and have broad

distributions of sizes. Following a bottleneck, alleles will be lost due to drift, but the allele size ranges may remain large. SD accounts for the clustered-ness of the distribution and is not sensitive to rare alleles. This means that while *M* responds quickly to sudden changes in N_e, SD reacts slowly as the overall distribution of alleles and ranges change over time. In our simulations we found that *M* both declined and recovered quickly, and exceeded the original levels even prior to population recovery. Meanwhile SD responded more slowly and did not increase until after the population began to increase and did not approach pre-bottleneck levels after 1,000 generations after full N_e recovery (Supplementary Figure 5b). Taken together, these divergent behaviours appear to be very useful, as a population displaying both high *M* and low SD may be a meaningful indicator of a bottleneck in a population that violates the strict SMM.

Using this approach, we found that *P.t. verus* displayed higher *M* than the rest of the species and lower SD (Supplementary Figure 6a – d). Supplementary Figure 6d effectively displays the distribution of our sampling locations based on the ratio of SD to *M*. Populations that tend toward the lower right corner of plot have a low SD and high *M*. We see that *P.t. verus* (with the exception of Mt. Sangbé, number 12, which is itself divergent among *P.t. verus*) diverges from most of the rest of the species. With its relatively high *M* and low SD, *P.t. verus* may be in process of overshooting its pre-bottleneck level of *M*, while not yet recovering SD, suggesting that a bottleneck occurred relatively recently. We also see that two *P.t. schweinfurthii* communities located in western Uganda, Budongo (45) and Ngogo (46), also tend toward the lower right hand side of the plot, indicating a possible signal of a relatively recent bottleneck.

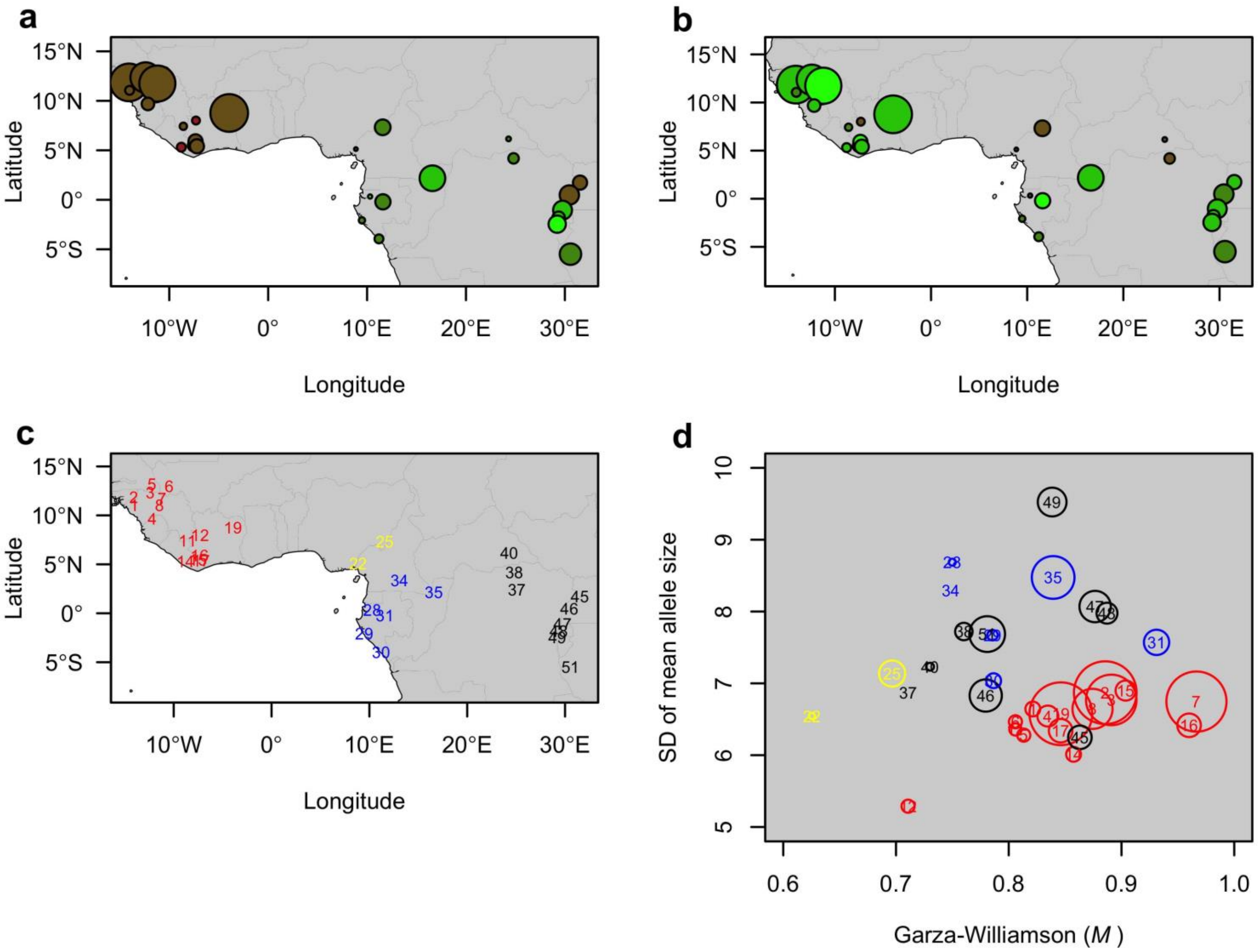

Supplementary Figure 6. Standard deviations (SD) of mean allele sizes and Garza-Williamson indices (*M*) among sampling locations. (**a**) A map of SD calculated among sampling locations in our dataset. Brown are sampling locations characterized by low SD values and green by high SD values. Samples from the *Pan troglodytes verus* range display relatively low SD values compared to the rest of the species. Two northeastern populations (Budongo and Ngogo) also display low SD values. (**b**) A map of *M* calculated among sampling locations in our dataset. Brown are sampling locations characterized with low relative levels of *M* and green are high levels. Samples from the *P.t. verus* range display slightly higher overall *M* than the rest of the species. In (a) and (b), circle diameter is correlated with sample size. Together these results suggest that *P.t. verus* has undergone a recent population bottleneck. (**c**) Chimpanzee subspecies populations categorized by colour: *Pan troglodytes verus* (red), *P.t. ellioti* (yellow), *P.t. troglodytes* (blue) and *P.t. schweinfurthii* (black). (**d**) Plot of *M* and SD among sampling

locations with a minimum of six individuals typed. Diameters of circles correlate with sample size. Populations displaying high *M* and low SD (lower right corner of plot) suggest a recent bottleneck event. Sampling locations 45 and 46 (Budongo and Ngogo) also display evidence of a recent population bottleneck.

We stress that many of the loci used in the present study do not follow strict SMM, therefore we cautiously offer these results. Nevertheless, we are comparing *M* within a single species in which the violations of strict SMM are similar across all subpopulations, i.e., per-locus overestimations of *M* are expected to be relatively consistent among populations and therefore comparable. However, lower *M* values in our dataset occur in the *ETS* populations, which are known to be more genetically diverse, and are therefore more at risks of inflated *M* due to having higher allele density per locus (leading to more potential for violations of strict SSM). In contrast, *P.t. verus* displayed higher *M* despite a paucity of allelic diversity. In sum, these factors and results are not at risk to the inherent limitations posed by violations of strict SMM in these analyses and may point toward a reliable signal of a widespread bottleneck event across *P.t. verus*, as well as in some local populations.

Spatially explicit analyses (EEMS)

Uniting spatial and genetic data provides distributional context to diversity and affords the use of unbalanced and heterogeneous sampling that should otherwise be avoided in spatially agnostic methods. We employed the spatially explicit software, Estimated Effective Migration Surfaces (EEMS)[36], to locate significant deviations from clinal variation in our data. As with all other analyses, we only included individuals typed at between seven and fourteen loci, to minimize noise and to maximize genetic data in the analyses. In tests of the minimum number of loci necessary to obtain the overall

observed pattern in the full dataset, we found that using the four most polymorphic loci produced the same general pattern, but without any areas of significant barriers, owing to both a decrease in the number of sampled individuals analysed and overall limitation of genetic data. Meanwhile, increasing the minimum to eight loci did not have any meaningful effect on the overall observed patterns. This indicates that using seven or more markers was sufficient for our analyses, and that adding more loci would be limited to improving detail in local patterns and not affect our overall conclusions. The EEMS analysis provides several diagnostic outputs to validate datasets after MCMC convergence (See main text for analysis parameters)[36]. The model was a good fit to our dataset (fitted versus observed genetic dissimilarity; adjusted $r^2 = .381$, $p < 0.001$; Supplementary Figure 7a). However, it was apparent that there were some influences associated with outliers, in particular, the presence of a conspicuous tail with a negative slope in the *P.t. verus* – *P.t. shcweinfurthii* comparisons on the right side of the plot. Since the points that constituted the tail were located at the extreme right side of the plot where between-deme distances were greatest, it was clear that this pattern was mostly driven by *P.t. verus* – Issa comparisons. To investigate this, we highlighted all comparisons involving Issa and found that the tail was nearly entirely composed of *P.t. verus* – Issa pairs (Supplementary Figure 7b). We also fitted a function for all between-demes comparisons involving Issa, which resulted in a horizontal line, indicating that this population was divergent from all sites regardless of distance. We had similar observations in our pairwise F'_{ST} analyses (Supplementary Figure 3b, 4a), further distinguishing the Issa population from the rest of our sampling locations. Finally, we

found that simply removing Issa from the dataset considerably improved model fit (adjusted $r^2 = 0.435$, $p < 0.001$).

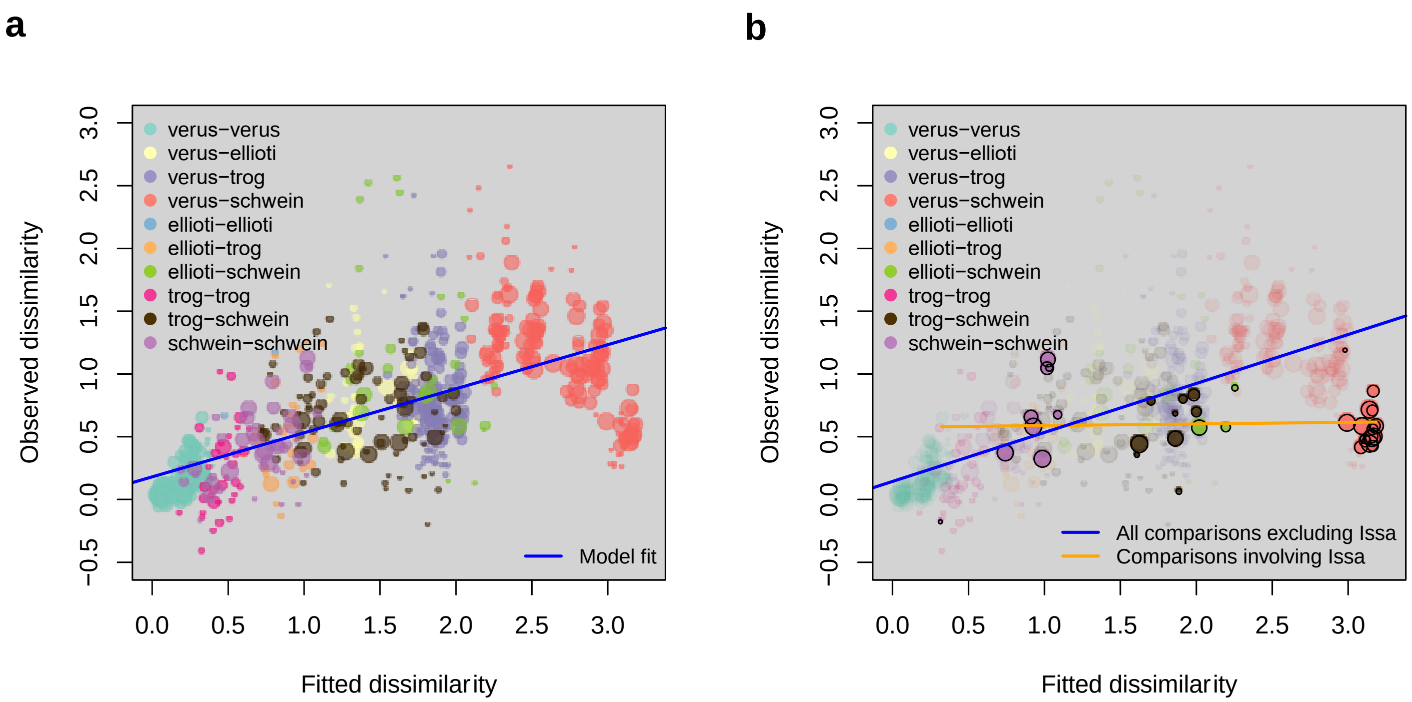

Supplementary Figure 7. Between-demes test of model fit. (**a**) A between-demes plot of fitted versus observed dissimilarity. The points are differentiated by subspecies-comparison categories and distinguished by color. The diameter of the points is proportional to the combined sizes of the paired demes being compared. The blue line is the fitted model (adjusted $r^2 = 0.381$). The most notable pattern in this plot is the negative slope of *Pan troglodytes verus – P.t. schweinfurthii* comparisons, which was likely, at least in part, driven by an outlier. (**b**) Fitted versus observed dissimilarity plot with Issa highlighted in between-demes comparisons. Points are as in **a**, except with Issa comparisons highlighted. The blue line is the model fitted to all comparisons excluding Issa (adjusted $r^2 = 0.435$, $p < 0.001$) and the orange line is the model fitted to all Issa pairwise comparisons. In pairwise F'_{ST} comparisons, the Issa population was very unusual in that they were equally differentiated from all other populations regardless of distance or subspecies. A similar pattern is depicted in **b** and, in part, explains the presence of the conspicuous tail on the right side of the plot.

In analyses of our entire dataset, we identified several locations in our sampling range in which significant barriers (posterior probability >95%) were present (Fig. 3a – c). These barriers appeared to be localized (between neighboring sites), and were likely a result of site-level environmental pressures. One such barrier was associated with Mt. Sangbé (Côte d'Ivoire), which exhibited a high proportion of first-order relatives (Supplementary Table 1.5) and is a population that has recently experienced considerable, if not complete, isolation from neighboring populations[17] another barrier was associated with the Gishwati (Rwanda) population. The Gishwati chimpanzees experienced a severe decline in the last two and half decades stemming from the fallout of the Rwandan genocide, reducing over 1,000 km^2 of forest to 9 km^2 in recent years[37]. The current chimpanzee population at Gishwati is estimated to be between19 and 29 individuals[38] and we identified a minimum of 24 individuals in this study. Additionally, along with Nyungwe, Gishwati is the nearest population to Issa, one of three consistent outliers in this study, and, importantly, equally differentiated from all sampling locations regardless of distance. The final significant barrier was between Mbe and Gashaka (Nigeria). This region has steep elevational gradients that may significantly restrict connectivity between these populations, with considerable differences in climate and habitat type. However, previous studies identified gene flow between similar regions of the *P.t. ellioti* range[28]. Additionally, as in Gishwati/Issa, Mbe is the closest in proximity to the highly differentiated Gashaka population. Notably, all of these barriers appeared to be attributable to localized differentiation rather than signals of reduced continuity between major populations or at the subspecies level. To assess these issues, we

subsequently removed these sampling locations and reanalyzed the resulting data (Supplementary Figure 8a – c).

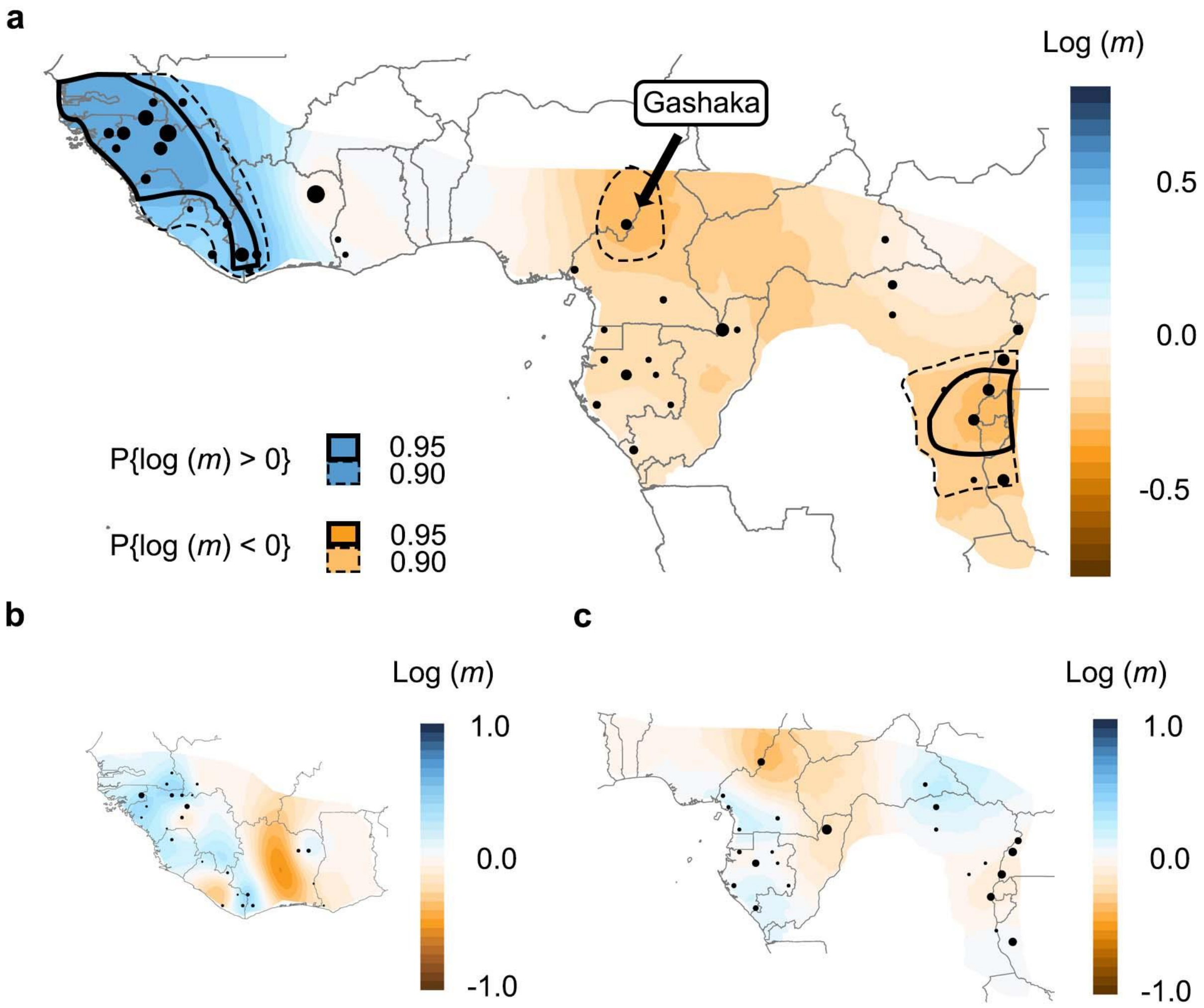

Supplementary Figure 8. EEMS maps of relative effective migration rates excluding outlier populations. (**a**) EEMS map of all four subspecies populations showing the effects on effective migrations rates (*m*) when excluding Mt. Sangbé, Mbe and Gishwati from the whole-species analysis. (**b**) EEMS map of the *P.t. verus* range showing the effect on effective migration (*m*) rates when excluding Mt. Sangbé from the analysis. (**c**) EEMS map of the *P.t. ellioti – P.t. troglodytes – P.t. schweinfurthii* (collectively *ETS*) range showing the effect on (*m*) of excluding Mbe and Gishwati from the analysis. *m* rates are mean centred and Log_{10} transformed. Locations within dashed lines indicate areas where the posterior probability of *m* > 0 exceeds 90%. Locations within solid lines indicate areas where the posterior probability of *m* > 0 is 95%. Note that no areas are significantly differentiated in (b) or (c) when each population, *P.t. verus* or *ETS*, is analysed independently and their influence on each other is removed.

An important consideration for this analysis is that it relies on a relativistic approach, thus extreme differences in effective population sizes, *e.g.*, between *P.t. verus* and *ETS*, will lead to over- and underestimation of posterior probabilities. In the case of our data, the high *m* rates in *P.t verus* cause areas in *ETS* to appear to have significantly low relative *m* rates. For this reason, it is necessary to also analyse these populations separately. At the full species scale, the barrier located in the range of Gishwati still had a 95% posterior probability of lower than average connectivity and the barrier associated with Mbe and Gashaka decreased from 95% to 90% (Supplementary Figure 8a). Upon exclusion of Mt. Sangbé, the results observed at the species scale remained similar, but a barrier signal is evident when analysing *P.t. verus* on its own (Supplementary Figure 8b). This signal of historical discontinuity suggests an additional source of reduced connectivity across Côte d'Ivoire.

Excluding Mbe and Gishwati from the *ETS* EEMS analysis removed the significance of barriers associated with these sites (Supplementary Figure 8c). These regions still displayed some weak signals of reduced connectivity relative to the average rate, suggesting the possibility of diminished historical connectivity in these areas, but well within rates observed elsewhere in *ETS*.

The loss of the significant barriers when excluding the sites that were associated with them is strong evidence that the discontinuity we observed was driven by localized differentiation. That these sites are genetically distinct from their neighbours, points to local effects and how strongly they influence site-level differentiation, and removing only

three outliers from our dataset eliminated all significant signals of discontinuity in these analyses.

Supplementary references

1. Arandjelovic, M. *et al.* Two-step multiplex polymerase chain reaction improves the speed and accuracy of genotyping using DNA from noninvasive and museum samples. *Mol. Ecol. Resour.* **9,** 28–36 (2009).
2. McCarthy, M. S. *et al.* Genetic censusing identifies an unexpectedly sizeable population of an endangered large mammal in a fragmented forest landscape. *BMC Ecol.* **15,** 21 (2015).
3. Di Rienzo, A. *et al.* Mutational processes of simple-sequence repeat loci in human populations. *Proc. Natl. Acad. Sci. U.S.A.* **91,** 3166–3170 (1994).
4. Taberlet, P. *et al.* Reliable genotyping of samples with very low DNA quantities using PCR. *Nucleic Acids Res.* **24,** 3189–3194 (1996).
5. Brinkmann, B., Klintschar, M., Neuhuber, F., Hühne, J. & Rolf, B. Mutation rate in human microsatellites: influence of the structure and length of the tandem repeat. *Am. J. Hum. Genet.* **62,** 1408–1415 (1998).
6. Ashley, C. T. & Warren, S. T. Trinucleotide repeat expansion and human disease. *Annu. Rev. Genet.* **29,** 703–728 (1995).
7. Rubinsztein, D. C. *et al.* Microsatellite evolution — evidence for directionality and variation in rate between species. *Nat. Genet.* **10,** 337–343 (1995).
8. Kimura, M. & Crow, J. F. The number of alleles that can be maintained in a finite population. *Genetics* **49,** 725–738 (1964).
9. Ohta, T. & Kimura, M. A model of mutation appropriate to estimate the number of electrophoretically detectable alleles in a finite population. *Genet. Res.* **22,** 201–204 (1973).
10. Schoske, R. The design, optimization and testing of Y chromosome short tandem repeat megaplexes. 1–255 (American University, 2003).
11. Lonsinger, R. C. & Waits, L. P. ConGenR: rapid determination of consensus genotypes and estimates of genotyping errors from replicated genetic samples. *Conserv. Genet. Resour.* **7,** 841–843 (2015).
12. Kalinowski, S. T., Taper, M. L. & Marshall, T. C. Revising how the computer program CERVUS accommodates genotyping error increases success in paternity assignment. *Mol. Ecol.* **16,** 1099–1106 (2007).
13. Chapuis, M.-P. & Estoup, A. Microsatellite null alleles and estimation of population differentiation. *Mol. Biol. Evol.* **24,** 621–631 (2006).
14. Sinnock, P. The Wahlund effect for the two-locus model. *Amer. Nat.* **109,** 565–570 (1975).
15. Csilléry, K. *et al.* Performance of marker-based relatedness estimators in natural populations of outbred vertebrates. *Genetics* **173,** 2091–2101 (2006).
16. Van Horn, R. C., Altmann, J. & Alberts, S. C. Can't get there from here: inferring kinship from pairwise genetic relatedness. *Anim. Behav.* **75,** 1173–1180 (2008).
17. Marchesi, P., Marchesi, N., Fruth, B. & Boesch, C. Census and distribution of chimpanzees in Côte D'Ivoire. *Primates* **36,** 591–607 (1995).
18. Pritchard, J. K., Wen, X. & Falush, D. Documentation for *structure* version 2.3 software: Version 2.3. 1–39 (2009).
19. Fünfstück, T. *et al.* The sampling scheme matters: *Pan troglodytes troglodytes* and *P. t. schweinfurthii* are characterized by clinal genetic variation rather than a

strong subspecies break. *Am. J. Phys. Anthropol.* **156,** 181–191 (2014).
20. Fischer, A. *et al.* Bonobos fall within the genomic variation of chimpanzees. *PLoS ONE* **6,** e21605 (2011).
21. Frantz, A. C., Cellina, S., Krier, A., Schley, L. & Burke, T. Using spatial Bayesian methods to determine the genetic structure of a continuously distributed population: clusters or isolation by distance? *J. Appl. Ecol.* **46,** 493–505 (2009).
22. Kalinowski, S. T. The computer program STRUCTURE does not reliably identify the main genetic clusters within species: simulations and implications for human population structure. *Heredity* **106,** 625–632 (2010).
23. Fischer, A., Pollack, J., Thalmann, O., Nickel, B. & paabo, S. Demographic history and genetic differentiation in apes. *Curr. Biol.* **16,** 1133–1138 (2006).
24. de Manuel, M. *et al.* Chimpanzee genomic diversity reveals ancient admixture with bonobos. *Science* **354,** 477–481 (2016).
25. Pritchard, J. K., Stephens, M. & Donnelly, P. Inference of population structure using multilocus genotype data. *Genetics* **155,** 945–959 (2000).
26. Evanno, G., Regnaut, S. & Goudet, J. Detecting the number of clusters of individuals using the software structure: a simulation study. *Mol. Ecol.* **14,** 2611–2620 (2005).
27. Arandjelovic, M. *et al.* Effective non-invasive genetic monitoring of multiple wild western gorilla groups. *Biol. Conserv.* **143,** 1780–1791 (2010).
28. Mitchell, M. W., Locatelli, S., Clee, P. R. S., Thomassen, H. A. & Gonder, M. K. Environmental variation and rivers govern the structure of chimpanzee genetic diversity in a biodiversity hotspot. **15,** 1–13 (2015).
29. Meirmans, P. G. & Hedrick, P. W. Assessing population structure: F_{ST} and related measures. *Mol. Ecol. Resour.* **11,** 5–18 (2010).
30. Excoffier, L. & Lischer, H. E. L. Arlequin suite ver 3.5: a new series of programs to perform population genetics analyses under Linux and Windows. *Mol. Ecol. Resour.* **10,** 564–567 (2010).
31. Mantel, N. The detection of disease clustering and a generalized regression approach. *Cancer Res.* **27,** 209–220 (1967).
32. Oksanen, J., Blanchet, F. G., Friendly, M., Kindt, R., Legendre, P., McGlinn, D., Minchin, P. R., O'Hara, R. B., Simpson, G. L., Solymos, P., Stevens, M. H. H., Szoecs, E., & Wagner, H. (2019). vegan: Community Ecology Package. R package version 2.5-6. https://CRAN.R-project.org/package=vegan
33. Meirmans, P. G. The trouble with isolation by distance. *Mol. Ecol.* **21,** 2839–2846 (2012).
34. Slatkin, M. Isolation by distance in equilibrium and non-equilibrium populations. *Evol.* **47,** 264–279 (1993).
35. Garza, J. C. & Williamson, E. G. Detection of reduction in population size using data from microsatellite loci. *Mol. Ecol.* **10,** 305–318 (2001).
36. Petkova, D., Novembre, J. & Stephens, M. Visualizing spatial population structure with estimated effective migration surfaces. *Nat. Genet.* **48,** 94–100 (2015).
37. Plumptre, A. J., Masozera, M. K. & Vedder, A. *The impact of civil war on the conservation of protected areas in Rwanda*. 1–34 (Biodiversity Support Program, 2001).
38. Chancellor, R. L., Langergraber, K. E., Ramirez, S., Rundus, A. S. & Vigilant, L.

Genetic sampling of unhabituated chimpanzees (*Pan troglodytes schweinfurthii*) in Gishwati Forest Reserve, an isolated forest fragment in western Rwanda. *Int. J. Primatol.* **33,** 479–488 (2012).
