# Supplementary material for: Recent genetic connectivity and clinal variation in chimpanzees
Source: Commun Biol. 2021 Mar 5;4:283. doi: 10.1038/s42003-021-01806-x (PMC7935964; doi:10.1038/s42003-021-01806-x)
Supplement: Supplementary file 2 — Reporting Summary [file 42003_2021_1806_MOESM2_ESM.pdf]

Corresponding author(s): Jack D. Lester and Mimi Arandjelovic

Last updated by author(s): January 14, 2021

Reporting Summary

Nature Research wishes to improve the reproducibility of the work that we publish. This form provides structure for consistency and transparency in reporting. For further information on Nature Research policies, see our Editorial Policies and the Editorial Policy Checklist.

Statistics

For all statistical analyses, confirm that the following items are present in the figure legend, table legend, main text, or Methods section.

n/a	Confirmed	
☐	☒	The exact sample size (*n*) for each experimental group/condition, given as a discrete number and unit of measurement
☐	☒	A statement on whether measurements were taken from distinct samples or whether the same sample was measured repeatedly
☐	☒	The statistical test(s) used AND whether they are one- or two-sided<br>*Only common tests should be described solely by name; describe more complex techniques in the Methods section.*
☐	☒	A description of all covariates tested
☐	☒	A description of any assumptions or corrections, such as tests of normality and adjustment for multiple comparisons
☐	☒	A full description of the statistical parameters including central tendency (e.g. means) or other basic estimates (e.g. regression coefficient) AND variation (e.g. standard deviation) or associated estimates of uncertainty (e.g. confidence intervals)
☐	☒	For null hypothesis testing, the test statistic (e.g. F, t, r) with confidence intervals, effect sizes, degrees of freedom and P value noted<br>*Give P values as exact values whenever suitable.*
☐	☒	For Bayesian analysis, information on the choice of priors and Markov chain Monte Carlo settings
☐	☒	For hierarchical and complex designs, identification of the appropriate level for tests and full reporting of outcomes
☐	☒	Estimates of effect sizes (e.g. Cohen's d, Pearson's r), indicating how they were calculated

Our web collection on statistics for biologists contains articles on many of the points above.

Software and code

Policy information about availability of computer code

Data collection	No custom software or code was used
Data analysis	No custom software or code was used

For manuscripts utilizing custom algorithms or software that are central to the research but not yet described in published literature, software must be made available to editors and reviewers. We strongly encourage code deposition in a community repository (e.g. GitHub). See the Nature Research guidelines for submitting code & software for further information.

Data

Policy information about availability of data

All manuscripts must include a data availability statement. This statement should provide the following information, where applicable:

- Accession codes, unique identifiers, or web links for publicly available datasets
- A list of figures that have associated raw data
- A description of any restrictions on data availability

The datasets generated during and analysed during the current study are available from the corresponding author on reasonable request.

Field-specific reporting

Ecological, evolutionary & environmental sciences study design

All studies must disclose on these points even when the disclosure is negative.

Study description	We analysed a broad scale and dense genetic sampling of chimpanzees across their geographic range to assess population structure and estimated effective migration rates.
Research sample	We non-invasively collected 5449 fecal samples from wild chimpanzees from 55 either temporary or long-term research sites over a period of 1 to 2 years.
Sampling strategy	We attempted to collect a minimum of 200 genetic samples from each location. This is roughly three times greater than the mean community size in chimpanzees. This target number provides the best possible sampling and genotyping success given our time frame.
Data collection	Samples were collected opportunistically when encountered during line transect surveys and opportunistically.
Timing and spatial scale	Samples were collected between 1 to 2 years from each sampling location in most cases. We also accepted opportunistically-collected samples from collaborators in remote locations. Sampling began in some locations in 2010 and ceased in December 2018. Sampling occurred in 18 countries across the entire range of the study species.
Data exclusions	We only excluded samples that were likely contaminated or did not genetically appear to be originating from a chimpanzee.
Reproducibility	For each sample we performed all PCR amplifications in triplicate to ensure over 99.9% certainty of identity. We accounted for the presence of null alleles, gentotyping error, and false alleles.
Randomization	Randomization was not relevant for our study, but we grouped samples from different field sites for both DNA isolation and PCR amplification steps to test for and safeguard against batch effect.
Blinding	Blinding was not relevant for our study, however we employed negatives for both DNA isolation and PCR amplification steps as a measure to test for the presence of contamination.

Did the study involve field work? ☒ Yes ☐ No

Field work, collection and transport

Field conditions	Sampling took place in 18 Equatorial African countries, in forests, anthropogenic landscapes, montane forests, rainforests, mixed-deciduous forest, and mixed mosaic habitat, comprising savannah and gallery forests, across full annual seasonal variation in ecological and climate conditions.
Location	Sampling took place at 55 sampling localities across Equatorial Africa where chimpanzees are extant. This encompassed a region of thousands of miles from East to West Africa including a variety of geographical areas and habitat types.
Access & import/export	Data were collected in compliance with all local, national and international laws in the 18 countries in which data were collected. When required, import and export permits were obtained from the appropriate government authorities prior to sample shipment.
Disturbance	Samples were collected opportunistically and every precaution was taken to avoid encounters with chimpanzees as well as other wild animals. Temporary field sites were returned to their natural state following the conclusion of data collection and all supplies and consumables were returned to Leipzig, Germany.

Reporting for specific materials, systems and methods

We require information from authors about some types of materials, experimental systems and methods used in many studies. Here, indicate whether each material, system or method listed is relevant to your study. If you are not sure if a list item applies to your research, read the appropriate section before selecting a response.

Materials & experimental systems

n/a	Involved in the study	
☒	☐	Antibodies
☒	☐	Eukaryotic cell lines
☒	☐	Palaeontology and archaeology
☐	☒	Animals and other organisms
☒	☐	Human research participants
☒	☐	Clinical data
☒	☐	Dual use research of concern

Methods

n/a	Involved in the study	
☒	☐	ChIP-seq
☒	☐	Flow cytometry
☒	☐	MRI-based neuroimaging

Animals and other organisms

Policy information about studies involving animals; ARRIVE guidelines recommended for reporting animal research

Laboratory animals	The present study did not involve laboratory animals
Wild animals	Animals, including the study species, were avoided under all circumstances. Sampling was opportunistic and non-invasive.
Field-collected samples	Samples were preserved in the field using a two-step ethanol/silica approach to achieve full dessication, then stored in a sealed Zargus box in a cool location, but was unrefrigerated for up to 2 years prior to transfer to Leipzig, Germany. Upon arrival samples were stored at -20 for up to 6 years until DNA extraction. DNA extracts were then stored at -80 until PCR amplification.
Ethics oversight	Data for this study did not involve animal experiments, captive animals, or human participants. All data were collected entirely non-invasively; fecal sample collection involved no contact with or disturbance to the chimpanzees under study.

Note that full information on the approval of the study protocol must also be provided in the manuscript.
